# Energy‐Resolved Femtosecond Dynamics of Plasmon‐Induced Hole Injection at Au/GaN Heterointerfaces

**DOI:** 10.1002/advs.202523801

**Published:** 2026-03-30

**Authors:** Yuying Gao, Yuxin Xie, Jonathan Diederich, Christian Höhn, Klaus Schwarzburg, Fengtao Fan, Can Li, Roel van de Krol, Dennis Friedrich

**Affiliations:** ^1^ Institute for Solar Fuels Helmholtz‐Zentrum Berlin für Materialien und Energie GmbH Berlin Germany; ^2^ State Key Laboratory of Catalysis Dalian National Laboratory for Clean Energy Dalian Institute of Chemical Physics Chinese Academy of Sciences Dalian China; ^3^ Institut Für Chemie Technische Universität Berlin Berlin Germany

**Keywords:** electron energy distribution, interfacial hole transfer, plasmonic photocatalysis, ultrafast electron dynamics

## Abstract

Plasmon‐induced interfacial hole injection at metal/semiconductor heterointerfaces represents a complementary pathway to conventional hot electron transfer, thereby broadening the functional landscape of plasmonic systems for energy conversion and optoelectronic technologies. However, direct experimental visualization of the energy‐resolved femtosecond dynamics associated with this process remains elusive, as the intricate interfacial properties significantly complicate the underlying mechanism. Here, we reveal unprecedented spectral signatures of an ultrafast nonthermal hole transfer process at the Au/GaN heterointerface, occurring within 49 fs after plasmon excitation, on a timescale comparable to hot electron transfer. This process exhibits pronounced sensitivity to both excitation energy and light polarization, leading to a substantial reshaping of the low‐energy electron distribution near the Fermi level by enhancing the low‐energy electrons population and reducing the decay rate. Harnessing this ultrafast nonthermal hole transport results in a 14‐fold enhancement in hydrogen evolution performance, demonstrating a promising approach for tailoring interfacial charge dynamics and offering mechanistic insights to guide the rational design of advanced plasmonic materials and device architectures.

## Graphical abstract

We reveal the spectral signature of ultrafast nonthermal hot‐hole transfer at plasmonic heterointerfaces, which reshapes hot‐carrier relaxation dynamics and significantly enhances hydrogen evolution, providing a mechanistic basis for optimizing hot‐carrier utilization in photocatalysis.

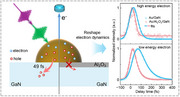

## 1 Introduction

Plasmonic nanostructures with distinctive and intriguing optical properties have attracted much attention in the fields of optoelectronic devices [1, 2, 3] and solar energy conversion [4, 5, 6]. Their functionality relies on surface plasmon resonance (SPR), which enhances light‐matter interactions and concentrates electromagnetic energy into nanoscale volumes. This localized near‐field effect enables the efficient generation of energetic hot carriers with distinct ultrafast relaxation dynamics [7, 8, 9] and non‐equilibrium energy distributions [10, 11]. The initial nonthermal energy distribution of hot carriers undergoes rapid thermalization via electron‐electron scattering on femtosecond timescales forming a quasi‐equilibrium state, followed by energy dissipation to the lattice through electron‐phonon coupling within a few picoseconds [12]. This implies that efficient separation and extraction of nonthermal carriers before fast thermalization is crucial for their successful application [13], particularly in hot‐carrier‐driven photochemical reactions [14, 15], as it directly governs how effectively energetic carriers participate in productive processes and determines photocatalytic selectivity [16, 17]. In this context, interfacial charge transfer in plasmonic metal/semiconductor heterostructures provides a critical pathway to circumvent ultrafast carrier relaxation by facilitating efficient charge separation [18, 19, 20]. Therefore, achieving a comprehensive understanding of the temporal and energetic behaviors of hot carriers, especially their transport dynamics across heterointerfaces, is essential for unlocking the full potential of plasmonic materials.

Numerous studies have extensively investigated plasmon‐induced ultrafast electron dynamics using transient absorption spectroscopy (TAS), with particular emphasis on processes such as electron generation [8], scattering [21, 22], and injection at heterointerfaces [18, 23, 24]. These studies have revealed that plasmon‐induced interfacial electron transfer in metal nanoparticles (NPs) is governed by particle size [25], structural symmetry [26], excitation wavelength [18], and interfacial electronic properties [8, 22]. Despite these advances, the complementary process of plasmon‐induced hole transfer remains comparatively underexplored. In particular, the injection of plasmonic hot holes from metal NPs into adjacent p‐type semiconductors represents a promising strategy for enhancing charge separation [20, 23]. This is particularly critical for plasmonic metals such as gold and copper, which generate hot holes with substantially higher energies than their electron counterparts [27], underscoring the need for a deeper understanding of hot hole injection mechanisms at metal/semiconductor interfaces. Recent TAS studies, for example, have shown that hot hole transfer from Au NPs to p‐GaN can significantly influence electron‐phonon coupling time and electronic temperature [23]. However, these optical probes primarily measure ensemble‐averaged cooling dynamics of quasi‐thermalized carrier populations, making it difficult to distinguish nonthermal carrier transfer from conventional thermalized transfer pathways. As a result, the microscopic mechanisms by which interfacial properties govern hole transfer dynamics and reshape electron energy distributions remain largely unclear. While theoretical calculations can predict the initial energy distribution of hot carriers generated by SPR excitation before inelastic relaxation in metal NPs [7, 10, 28], a quantitative description of electron energy distribution in the presence of interfacial hole transfer at chemically complex heterointerface has not yet been realized.

In this study, we investigate plasmon‐induced interfacial hot hole injection in Au/GaN heterostructures by combining surface photovoltage microscopy (SPVM) to resolve spatial charge distributions with time‐resolved two‐photon photoemission spectroscopy (tr‐2PPE) to capture ultrafast carrier dynamics across temporal and energetic scales. By systematically disentangling the energy‐resolved build‐up dynamics of photoelectron emission signals, we unambiguously identify an ultrafast nonthermal hole transfer process from Au NPs into the GaN valence band, occurring within 49 fs after SPR excitation. This ultrafast process leads to a more than order‐of‐magnitude (14‐fold) improvement in charge separation and utilization efficiency in photoelectrochemical systems, thereby providing a compelling proof‐of‐concept demonstration of hot‐hole‐driven plasmonic photocatalysis. Our findings establish detailed spectral and temporal insights into ultrafast hot‐hole injection and elucidate its critical role in modulating electron dynamics in plasmonic photocatalysts.

## 2 Results and Discussion

### 2.1 Characterization of Au/GaN

**FIGURE 1:**
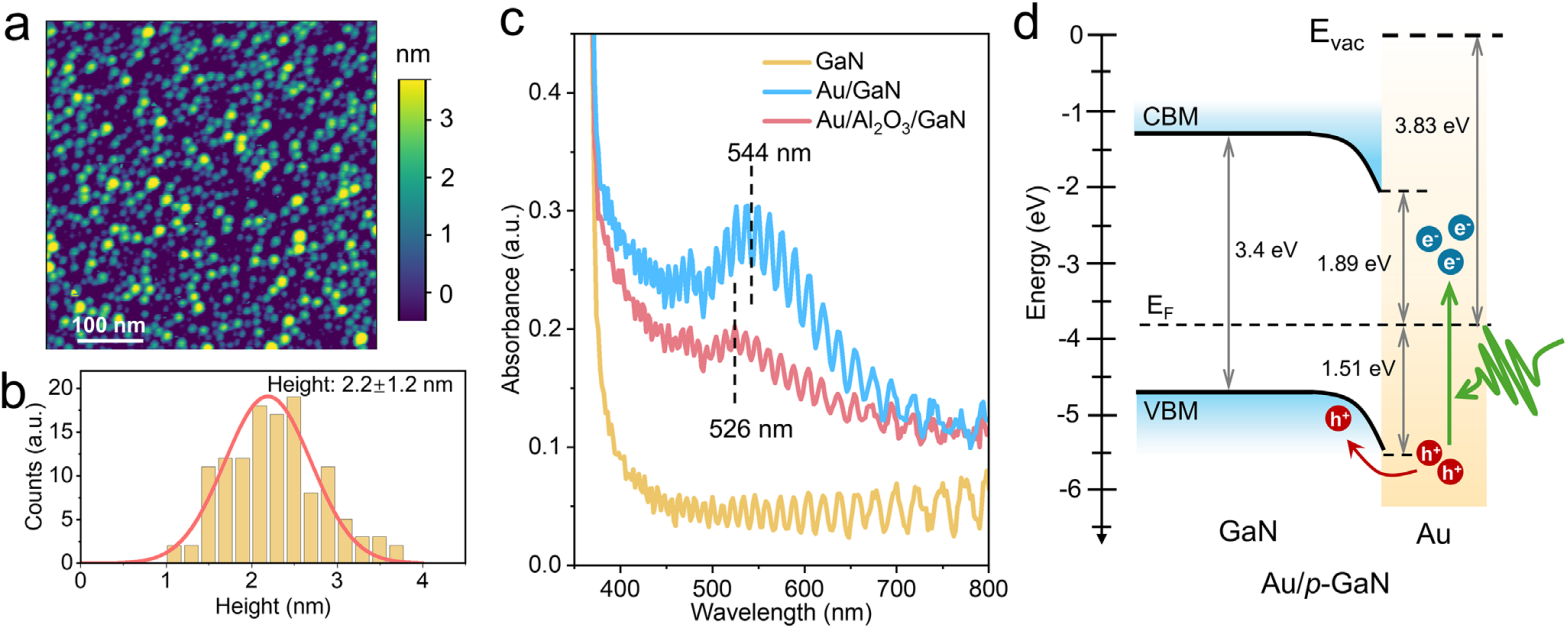
Characterization of the plasmonic Au/p‐GaN heterostructure. (a) AFM image of Au NPs on the p‐GaN substrate. (b) Height distribution of Au NPs on GaN substrate with a Gaussian fit. (c) UV‐vis absorption spectra of the Au/p‐GaN heterostructure, Au/Al_2_O_3_/GaN heterostructure, and the bare p‐GaN substrate. The absorption edge is visible for all samples at 365 nm due to the bandgap of GaN. A plasmonic absorption peak is located at 544 nm (2.28 eV) for the Au/p‐GaN heterostructure. The fringes are due to Fabry‐ Pérot interference. (d) Schematic of energy level alignment between Au NPs and the p‐GaN substrate, along with the expected plasmon‐induced hole transfer path across the heterostructure interface. Labels: E_F_, Fermi level; E_vac_, vacuum energy level; CBM, conduction band minimum; VBM, valence band maximum.

Gold NPs were deposited on p‐GaN wafers via electron beam evaporation in an ultra‐high vacuum (UHV) environment after surface cleaning of GaN substrate. Low‐energy electron diffraction (LEED) confirmed a reconstructed GaN surface with a characteristic (1 × 1) hexagonal pattern (Figure S1), indicating an ordered Ga adlayer on top of a Ga‐terminated surface [29]. Atomic force microscopy (AFM) image (Figure 1a) shows uniformly sized Au NPs randomly distributed on the smooth GaN surface. A typical height profile in AFM reveals Au NPs with a uniform height of 2.2 nm (Figure 1b). This direct contact between plasmonic components and semiconductor serving as a carrier extraction layer is crucial for efficient interfacial charge transfer in plasmon‐driven solar energy conversion [8, 20]. To systematically investigate interfacial charge transfer, we fabricated a control sample (Au/Al_2_O_3_/GaN) by inserting a 5‐nm‐thick Al_2_O_3_ interlayer via atomic layer deposition (ALD), while keeping the identical Au NPs size (Figures S2 and S3). Because of the wide bandgap (∼6.8 eV) and the Fermi level being far from the band edge of Al_2_O_3_ (Figure S4), plasmon‐induced charge injection at the Au/Al_2_O_3_/GaN interface is effectively suppressed [23]. Additionally, due to the relatively thick Al_2_O_3_ layer used here, hot carrier tunnelling across the interface can be neglected, as this process typically occurs when the dielectric layer thickness is below 2 nm [30].

Optical absorption spectra of the Au/GaN (Figure 1c) exhibit a well‐resolved SPR peak at 544 nm (2.28 eV) and a sharp absorption edge at 365 nm corresponding to the bandgap energy of GaN (3.4 eV). In contrast, the introduction of the Al_2_O_3_ layer between Au and GaN leads to a weaker blue‐shift of SPR peak to 526 nm due to the lower refractive index of Al_2_O_3_ compared to GaN. The insertion of a 5‐nm Al_2_O_3_ interlayer significantly reduces the SPR amplitude of the Au NPs. This suppression primarily arises from the lower surrounding dielectric constant (1.6 for Al_2_O_3_, 2.4 for GaN), which decreases both oscillator strength and near‐field enhancement. The spacer also decouples the NPs from the high‐index GaN substrate, weakening image‐charge interactions and substrate‐induced field confinement [31]. AFM analysis confirms that nanoparticle size and density remain unchanged (Figure 1a; Figure S3), and the resonance linewidth shows minimal broadening, indicating negligible damping from interface states. Therefore, the reduced SPR reflects mostly dielectric screening and weakened electromagnetic coupling, consistent with prior studies on plasmonic systems [32]. Such optical variations have negligible impact on subsequent hot‐electron relaxation behavior because of the power‐independent charge dynamics discussed later. The absorption fringes observed for all three samples originate from Fabry‐Pérot interference [23, 33]. The clear spectral separation between the SPR excitation and the GaN bandgap allows for selective tracking of plasmon‐induced charge separation mechanisms.

Figure 1d illustrates the schematic of energy band alignment of Au/GaN, determined from the optical absorption and valence band measurements. The bare p‐GaN surface shows substantial downwards band bending (∼2 eV), arising from the Fermi level pinning by Ga dangling bonds states near the conduction band minimum (CBM) (Figure S5), consistent with previous reports [34, 35]. Valence band spectroscopy shows a significant density of surface states above the valence band maximum (VBM), extending toward the Fermi level (Figure S5b). These states are attributed to shallow Mg‐acceptor dopants in p‐GaN [36], with a density approximately one order of magnitude lower than that of valence band. After Au NPs deposition, the VBM of GaN is 1.51 eV below the Fermi level (Figure S6), allowing only hot holes above this threshold (1.51 eV) to be injected into the GaN valence band. For the Au/GaN, the Fermi level is pinned by interface states at ∼0.19 eV below the mid‐gap position, with energy separation of 1.51 eV from the VBM and 1.89 eV from the CBM.

### 2.2 Imaging Plasmon‐Induced Hole Injection at Interface

**FIGURE 2:**
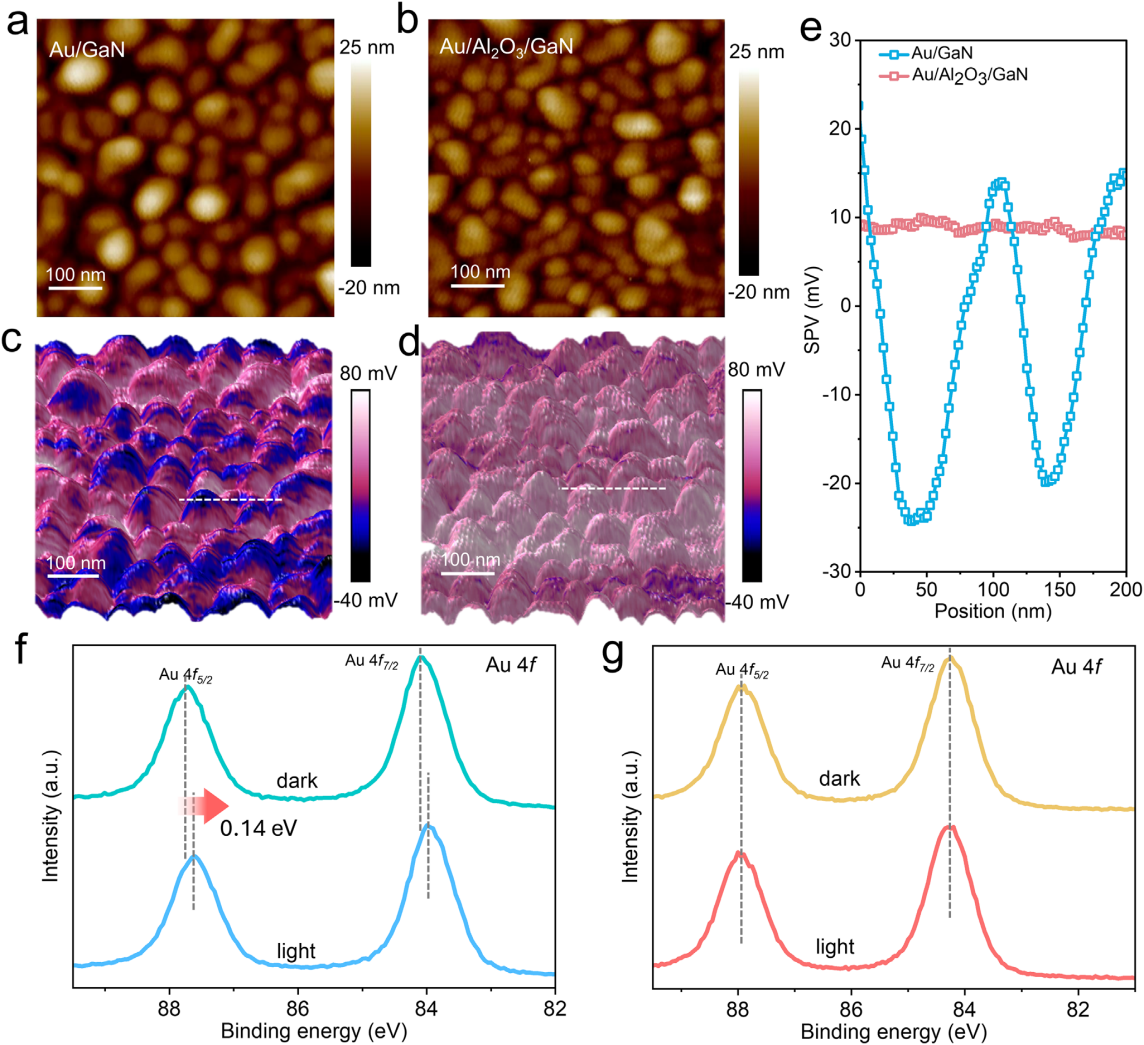
Demonstration of plasmon‐induced interfacial hole injection. (a,b) Surface topography for (a) Au/GaN and (b) Au/Al_2_O_3_/GaN samples. (c) 3D image showing the overlap of surface photovoltage (SPV) and surface topography for Au/GaN under SPR excitation. The surfaces of Au NPs show a clear negative SPV. (d) 3D image showing the overlap of SPV and surface topography for Au/Al_2_O_3_/GaN under SPR excitation. (e) SPV values extracted across dashed lines in c and d. (f,g) High‐resolution XPS spectra of Au 4*f* for (f) Au/GaN and (g) Au/Al_2_O_3_/GaN heterostructures in the dark and under light illumination (532 nm, 100 mW/cm^2^). The dashed grey lines serve as a guide to the eye. The red arrow indicates the shift in binding energy upon illumination.

We first employed surface photovoltage microscopy (SPVM) to directly map plasmon‐induced charge distribution with nanometer resolution (∼ 10 nm) at steady state. To better identify the single Au NPs, larger Au NPs with a diameter of approximately 16 nm were used for SPVM imaging (Figure 2a,b; Figure S7). Figure 2c,d show SPVM images of Au/GaN and Au/Al_2_O_3_/GaN, respectively, under SPR excitation using monochromatic light at 550 nm (5 mW/cm^2^). The SPV signal, representing the surface potential change upon illumination, shows a negative SPV on the Au NPs surface in Au/GaN. This indicates plasmon‐induced hole injection into the GaN, while plasmon‐excited electrons remain localized on the Au NPs. In contrast, no detectable SPV response is observed for Au/Al_2_O_3_/GaN under identical illumination conditions, consistent with negligible interfacial charge transfer. Quantitative analysis of SPV distributions reveals a negative signal of ‐20 mV at Au NPs in Au/GaN, confirming efficient plasmonic hole injection across the interface (Figure 2e).

High‐resolution X‐ray photoelectron spectroscopy (XPS) further confirms interfacial charge transfer under visible light illumination (532 nm, 100 mW/cm^2^), focusing on the Au 4*f*_5/2_ and 4*f*_7/2_ core‐level states [37]. As shown in Figure 2f,g, both levels on the Au/GaN surface shift by 0.14 eV toward lower binding energies upon illumination, whereas no such shift is observed for Au/Al_2_O_3_/GaN. Concurrent with the shift of Au 4*f*, the N 1*s* and Ga 2*p* levels in Au/GaN shift toward higher binding energies (Figure S8), indicating an increase in electron density in the Au NPs accompanied by a buildup of positive charge in the GaN substrate. Combined with the band alignment shown in Figure 1d, these spectral features provide compelling evidence for plasmon‐induced hole transfer from Au NPs to the GaN valance band.

### 2.3 Ultrafast Dynamics of Nonthermal Hole Transfer

To further elucidate the dynamics of interfacial hole transfer and its impact on hot electron relaxation, we performed femtosecond tr‐2PPE measurements to investigate the ultrafast electron dynamics at surfaces and interfaces with both temporal and energetic resolution [38, 39]. The principle of tr‐2PPE is based on a pump‐probe scheme utilizing two ultrashort laser pulses with a controlled time delay. As illustrated schematically in Figure S9a, an initial femtosecond pump pulse with a photon energy of 2.25 eV (550 nm) excites electrons from occupied states to unoccupied intermediate states in the Au NPs, while a time‐delayed probe pulse with a photon energy of 4.49 eV (276 nm) promotes these transient electrons above the vacuum level. By analyzing the kinetic energy of the emitted photoelectrons using an electron energy analyzer, we can reconstruct the energy distribution of the intermediate electronic states as a function of the delay time. The temporal resolution of our tr‐2PPE setup is determined to be ∼40 fs, allowing direct measurement of electron injection, relaxation, and recombination dynamics, which provides critical insights into the associated hole transfer processes. Both beams were p‐polarized and incident from the Au nanoparticle side at an angle of 45° relative to the sample surface, with the emitted electrons collected along the normal surface. Notably, the pump photon energy lies below the GaN bandgap, ensuring that the observed dynamics originate exclusively from plasmonic excitations in Au NPs rather than from direct GaN band‐to‐band transitions.

**FIGURE 3:**
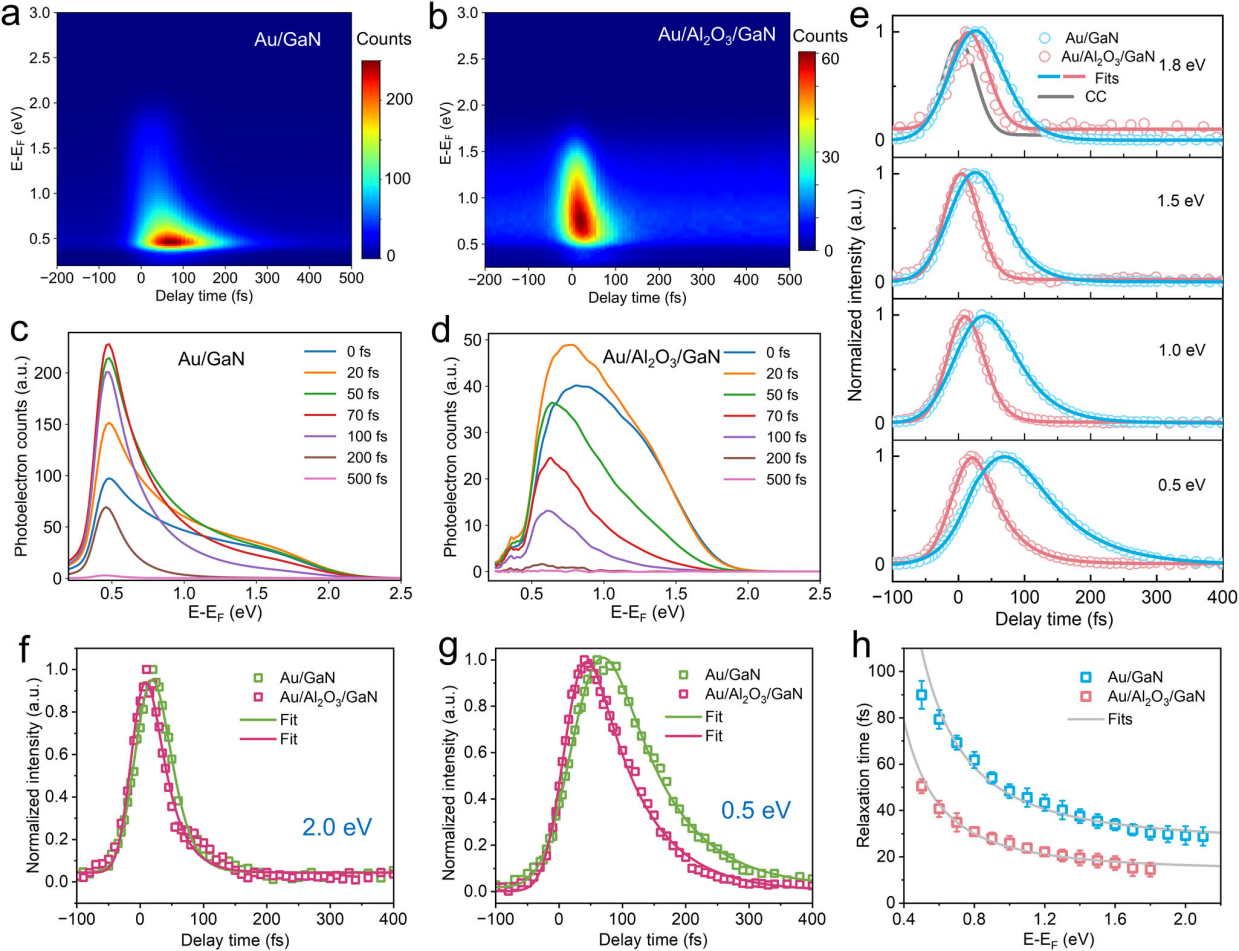
Observation of ultrafast nonthermal hole injection. (a,b) Representative pseudo‐color tr‐2PPE spectra as a function of pump‐probe delay time for (a) Au/GaN and (b) Au/Al_2_O_3_/GaN. All spectra were measured at a pump energy of 2.25 eV (550 nm) and a probe energy of 4.49 eV (276 nm). (c,d) Time‐dependent 2PPE counts as a function of energy relative to the Fermi level (E‐E_F_) for Au/GaN (c) and Au/Al_2_O_3_/GaN (d) at various pump‐probe delay times. (e) Energy‐resolved photoemission intensity as a function of pump‐probe delay time for Au/GaN and Au/Al_2_O_3_/GaN. The open circles are experimental data points, and the solid lines are fits to the equations described in Note S2. The gray line is the pump‐probe cross‐correlation obtained on a Cu (100) crystal surface. (f,g) Normalized 2PPE intensity as a function of pump‐probe delay time for Au/GaN and Au/Al_2_O_3_/GaN, obtained at electron energies of (f) 2.0 eV and (g) 0.5 eV. The spectra were measured at the pump energy of 2.64 eV (470 nm). The solid lines are fits to obtain time constants of hot electron relaxation. (h) Comparison of lifetimes (emission decay time constants) for Au/GaN and Au/Al_2_O_3_/GaN. The grey lines represent fits based on Fermi‐liquid theory.

Representative 2D pseudo‐color tr‐2PPE spectra for Au/GaN and Au/Al_2_O_3_/GaN are shown in Figure 3a,b. To depict the excited‐state energy distribution relative to the Fermi level, we present the energy of excited state as *E ‐ E_F_ = E_kin_ ‐ hv ‐ ϕ+W*, where *E_kin_* is the photoelectron kinetic energy, *ϕ* is the applied potential (0.5 V) between sample and photoelectron detector, *W* is the surface work function determined by UPS (Figures S6 and S9), and *hv* is the probe photon energy. The 4.49 eV probe corresponds to an electron inelastic mean free path of approximately 5 nm [40], which exceeds the ∼2.2 nm size of the Au NPs. This configuration minimizes inelastic scattering, ensuring that the measured spectra reliably reflect the intrinsic hot‐electron dynamics.

For both Au/GaN and Au/Al_2_O_3_/GaN samples, a central emission peak is observed at ∼20 fs pump‐probe delay, corresponding to multi‐photon photoemission from occupied states via virtual intermediate states. The linear dependence of the emission intensity on both pump and probe powers indicates that the observed signal originates from a one‐photon pump and one‐photon probe process in both systems (Figures S10 and S11). In addition, a time‐independent background is observed at ∼0.45 eV for Au/GaN and between 0.5 and 1.5 eV for Au/Al_2_O_3_/GaN, originating from one‐photon UV photoemission and two‐photon visible light photoemission from the Fermi level, respectively.

To isolate the transient spectral contributions, the steady‐state background was subtracted, yielding the time‐dependent spectra shown in Figure 3c,d at various pump‐probe delays. The observed 2PPE signal intensity reflects the photoemission yield of detectable electrons rather than the total hot‐electron population, thus the key comparison focuses on the rise and decay dynamics rather than absolute amplitude. The spectral feature at 0 fs clearly shows a nonthermal electron distribution extending up to E_F_+hν for both Au/GaN and Au/Al_2_O_3_/GaN, consistent with theoretical predictions [41]. As the delay time increases, high‐energy electrons relaxation results in a decrease in their population, accompanied by an increase in the population of low‐energy electrons. This indicates that photoemission originates primarily from Au NPs rather than GaN. Notably, the low‐energy electron population (centered around 0.5 eV) rises over about 70 fs on Au/GaN, whereas this same process occurs within only ∼20 fs in Au/Al_2_O_3_/GaN. This pronounced difference indicates distinct hot electron thermalization pathways in the presence of interfacial charge transfer, leading to a slower relaxation on Au/GaN.

To elucidate the origin of the distinct ultrafast charge dynamics, Figure 3e presents the rise and decay times of the emission signal at various electron energies relative to E_F_, together with the pump‐probe cross‐correlation (CC) measured on a Cu (100) surface. Both Au NP samples exhibit slower emission decay than the CC trace, confirming that hot electrons relaxation occurs on timescales exceeding the instrument's temporal resolution (∼40 fs). At higher electron energies (∼1.8 eV), the rise dynamics of the two samples are nearly identical, within the uncertainty of the CC. A similar trend is consistently reproduced under a different pump photon energy (2.64 eV, Figure 3f). However, as the electron energy decreases toward E_F_, an intriguing phenomenon emerges: the rise time (*τ_rise_*) in Au/GaN becomes noticeably slower than in Au/Al_2_O_3_/GaN, particularly for low‐energy electrons around 0.5 eV. Several mechanisms could in principle account for this delay, including plasmon dephasing [42], variations in hot electron generation rate [8], Auger scattering [43], or interfacial charge transfer [15]. However, no dependence of charge dynamics on pump fluences was observed for either high‐energy or low‐energy electrons in Au/GaN and Au/Al_2_O_3_/GaN samples (Figures S12 and S13), indicating that the rise times and relaxation kinetics are insensitive to the total excited carrier density. This robust power‐independent behavior confirms that the observed temporal delays do not arise from reduced plasmon excitation in the Al_2_O_3_‐spaced sample. Moreover, the nearly identical rise dynamics at high electron energies, together with the pronounced deviations observed only at low energies, exclude a purely optical origin, since reduced excitation would uniformly scale the carrier population rather than produce energy‐selective dynamic differences. We further note that, for the same nanoparticle size, the plasmon dephasing times for both samples are very similar, approximately 1.5 fs (Figure S14 and Note S1). Given that Au/GaN and Au/Al_2_O_3_/GaN share identical nanoparticle morphologies but differ in their ability to support interfacial charge transfer, the slower rise of low‐energy electrons in Au/GaN can be attributed primarily to the additional contribution of charge transfer channels [22]. In Au NPs, optical excitation creates correlated electron‐hole pairs such that the appearance of low‐energy electrons is necessarily accompanied by the presence of corresponding high‐energy holes. Thus, the slower rise of low‐energy electrons in Au/GaN reflects modified carrier thermalization dynamics rather than direct depletion of high‐energy electrons. Ultrafast hot‐hole extraction at the Au/GaN interface perturbs the early electron‐electron scattering‐mediated redistribution of carriers, thereby delaying the buildup of electrons near the Fermi level. Consequently, the temporal evolution of the low‐energy electron population serves as a sensitive probe for the extraction dynamics of their hole counterparts. Specifically, ultrafast extraction of high‐energy holes at the Au/GaN interface suppresses their participation in internal scattering cascades, thereby delaying the buildup of low‐energy electrons and providing direct evidence of nonthermal plasmonic hole injection, which is most pronounced at lower electron energies where deeper holes exhibit higher transfer probability [44]. The resulting modification of local electron occupancy and screening conditions accounts for the observed dynamics.

By performing a global fitting analysis of rise and decay dynamics (solid lines in Figure 3e, see details in Note S2), we extract an ultrafast plasmon‐induced hot hole transfer time of 49 ± 14 fs in the Au/GaN. This timescale represents a significant departure from the ∼200 fs transfer regime previously reported for similar interfaces using TAS [23]. These energy‐ and time‐resolved tr‐2PPE measurements provide unique insight into this process, directly revealing a nonthermal hole transfer that occurs on a timescale competing with internal thermalization pathways. Such a regime remains fundamentally inaccessible to conventional ensemble‐averaged techniques, which typically probe the relaxation of quasi‐thermalized carriers. This timescale is comparable to the reported lifetime of d‐band holes (∼70 fs) in Au NPs [45], indicating that interfacial injection occurs before the holes undergo significant energy loss through rapid scattering. This finding shifts the physical paradigm from thermalization cooling to a nonthermal injection, which is particularly critical at heterointerfaces with large Schottky barriers where only high‐energy, non‐thermalized carriers can be effectively extracted [33]. Furthermore, the charge transfer time exhibits a strong dependence on the excitation energy, shortening to 22 fs under interband excitation at 470 nm (Figure 3g). In this regime, interband transitions predominantly generate low‐energy electrons near the Fermi level along with deeper‐lying d‐band holes (Figure S15), which promote more efficient interfacial hole transfer on the femtosecond timescale. This pronounced acceleration emphasizes the role of the initial hot‐carrier energy distribution in governing the transfer efficiency and provides compelling support for a nonthermal hole transfer mechanism operating on femtosecond timescales.

The above quantitative analysis allows us to plot the lifetimes of hot electrons as a function of excited‐state energy E‐E_F_, as shown in Figure 3h. Consistent with Fermi liquid theory [46], the decay times increase with decreasing energy, in agreement with earlier reports [47]. However, the lifetimes measured in both Au NP systems are significantly shorter than those reported for Au thin films [46]. This deviation can be ascribed to differences in morphology and excitation conditions: the ultrasmall (∼2 nm) Au NPs used here are in intimate contact with the GaN substrate, where strong interfacial coupling enhances electron‐phonon interactions and accelerates relaxation, unlike in extended Au film. Additionally, our excitation wavelength is resonant with the SPR of Au NPs, producing a high density of non‐equilibrium carrier population [21] that intensifies many‐body scattering (e.g., electron‐electron interactions). In contrast, previous studies on Au films typically employed off‐resonant excitation, where such plasmonic effect was negligible, resulting in comparatively longer relaxation times. Interestingly, the electron relaxation rate is slower in Au/GaN than in Au/Al_2_O_3_/GaN. While similar lifetime extensions of plasmonic electrons have been observed in Au/TiO_2_ relative to Au/SiO_2_ in TAS studies [22], our tr‐2PPE measurements enable a level of mechanistic resolution inaccessible to ensemble optical probes. Specifically, the energy‐resolved nature of tr‐2PPE reveals that this lifetime extension is not a uniform thermal effect across the carrier distribution but is instead most pronounced for low‐energy electrons near E_F_. The absence of interfacial charge transfer channels in Au/Al_2_O_3_/GaN results in elevated electron temperatures, which enhances many‐body scattering events and thus accelerates electron relaxation [48]. By contrast, in Au/GaN, energy‐resolved tracking directly shows that selective extraction of high‐energy holes reshapes the residual carrier population. This decoupling of interfacial charge transfer from intrinsic relaxation establishes hole injection as an effective energy‐selective dissipation pathway, removing the most energetic carriers before extensive scattering occurs. Such a microscopic fingerprint of carrier redistribution provides direct evidence that nonthermal interfacial transfer governs the subsequent relaxation landscape, a detail that is fundamentally obscured in ensemble‐averaged optical measurements.

### 2.4 Polarization Dependence of Ultrafast Charge Transfer Dynamics

**FIGURE 4:**
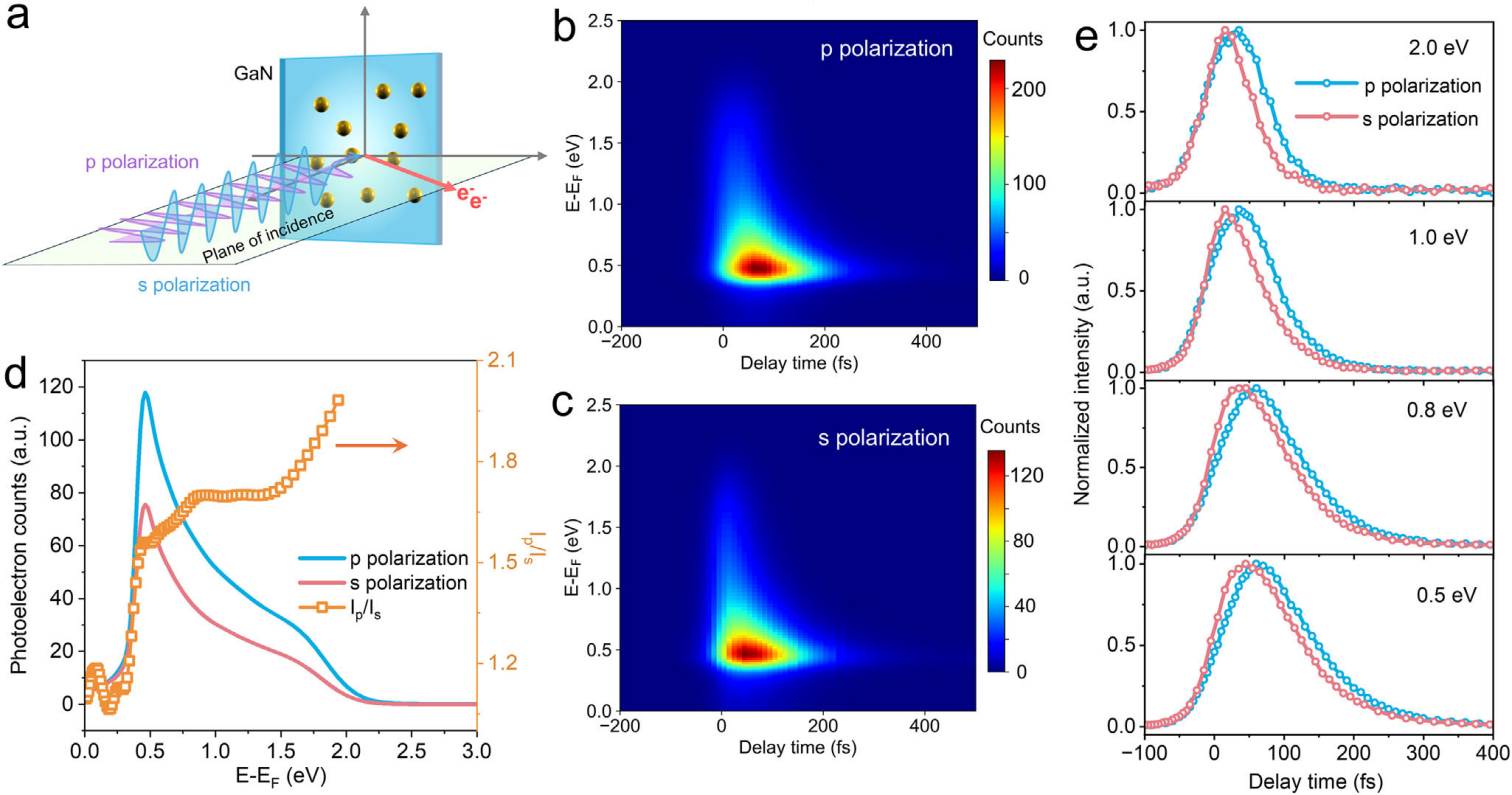
Polarization dependence of electron dynamics driven by interfacial nonthermal holes injection. (a) Schematic illustration of pump polarization‐dependent tr‐2PPE measurements. The red arrow indicates the emitted photoelectrons. (b,c) Background‐subtracted pseudo‐color tr‐2PPE spectra as a function of pump‐probe delay time for Au/GaN under (b) p polarization laser excitation and (c) s polarization laser excitation. (d) 2PPE spectra (left axis) for Au/GaN excited by p‐ and s‐polarized pulses (550 nm). The ratio of p‐polarized to s‐polarized photoemission is displayed on the right axis. (e) Experimental data of normalized energy‐resolved photoelectron emission signal as a function of pump‐probe delay time for Au/GaN excited by p‐ and s‐polarized pump laser.

Given the known polarization dependence of plasmonic excitations [26, 49], we further investigated the ultrafast charge transfer dynamics by recording tr‐2PPE spectra at different pump beam polarizations using a half‐wave plate (Figure 4a). The ultraviolet probe beam was kept at p‐polarization, while the visible pump beam was alternated between p‐ and s‐polarizations. As shown in Figure 4b,c, the photoemission signal is stronger under p‐polarized excitation than under s‐polarized excitation. The polarization dependence originates from the orientation of the electric field relative to the Au/GaN interface. For p‐polarized excitation, the incident field possesses a component normal to the interface, which promotes interfacial carrier coupling and facilitates the extraction of high‐energy holes from Au into GaN. In contrast, s‐polarized excitation produces an electric field parallel to the surface, resulting in weaker coupling to interfacial charge transfer pathways. Figure 4d shows the spectra recorded at 0 fs delay for both polarization conditions, along with the emission intensity ratio (I_p_/I_s_) as a function of energy relative to E_F_. Under p‐polarization, a stronger 2PPE signal is observed due to the enhanced localized electric field distribution and more efficient plasmon excitation [50]. Analysis of the normalized spectra reveals that p‐polarization preferentially increases the high‐energy electron population (Figure S16), leading to a higher I_p_/I_s_ ratio above ∼1.4 eV. Directly above the emission cut‐off at around 0.5 eV, I_p_/I_s_ is approximately 1.5, increasing to 1.7 at 0.9 eV and remaining nearly stable up to 1.5 eV. The observed decrease in the I_p_/I_s_ ratio for energies below 1.0 eV is attributed to the enhanced interfacial transfer of plasmonic holes under p‐polarized excitation, rather than to the direct excitation of the SPR. This enhancement arises because p‐polarized light generates a larger fraction of carriers with appropriate momentum for injection. Compared to Ag‐based counterparts [50, 51], the lower I_p_/I_s_ ratio primarily reflects the exclusive modulation of pump polarization in our measurements, combined with the intrinsically broader and more heavily damped plasmonic resonances of Au systems caused by its pronounced interband transitions in the visible spectral range.

As expected, comparison of energy‐sliced tr‐2PPE spectra under p‐ and s‐polarized excitation (Figure 4e) shows that the emission rise times are nearly identical at higher electron energies (>1.0 eV vs E_F_). However, the rise time under p‐polarization is delayed by 18 ± 6 fs relative to s‐polarization at electron energy of 0.5 eV. Given that the tr‐2PPE dynamics are independent of pump power (Figure S12) and that no analogous spectral features appear in the Au/Al_2_O_3_/GaN control sample under any excitation condition (Figure S17), this polarization dependence provides direct evidence that p‐polarized excitation enhances ultrafast nonthermal hole injection at the Au/GaN interface. This correlation between energy‐resolved dynamics and pump polarization demonstrates that the apparent slowing of state‐specific hot‐electron relaxation arises from interfacial hole extraction rather than a uniform thermalization effect, occurring well before the establishment of a Fermi‐Dirac distribution. Moreover, analysis of the decay kinetics further reveals that the electron lifetime at 0.5 eV vs E_F_ is extended by 12 ± 6 fs under p‐polarization compared to s‐polarization, reinforcing the conclusion that interfacial hole transfer modulates the subsequent hot electron relaxation dynamics.

The above results provide experimental insight into the critical role of polarization, not only in governing SPR excitation and shaping the initial energy distribution of hot electrons but also in facilitating ultrafast nonthermal hole injection. This is particularly significant because of the largely unexplored nature of photogenerated hole transport in plasmonic heterostructures, which is a more challenging pathway for charge separation due to the inherently shorter mean free path and lifetime of holes [52, 53]. Demonstrating polarization‐sensitive charge transfer pathways offers a new degree of freedom for engineering ultrafast charge dynamics, potentially enabling new opportunities to optimize charge extraction and enhance the efficiency of hot‐carrier‐driven reactions in plasmonic photocatalysis.

### 2.5 Photoelectrochemical (PEC) Water‐Splitting Performance

**FIGURE 5:**
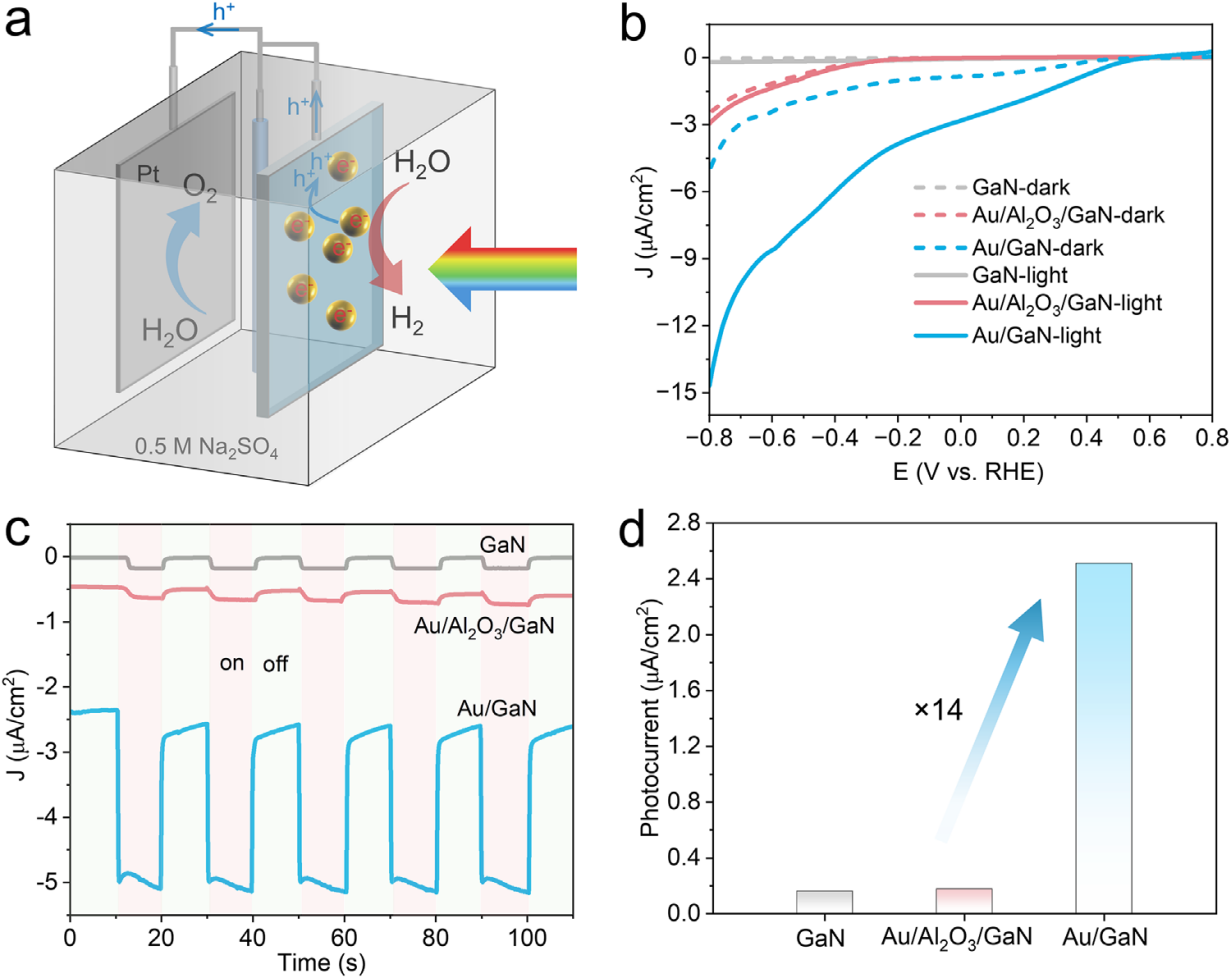
PEC water‐splitting performance. (a) Schematic diagram of photoelectrochemical setup for H_2_ evolution on plasmonic photocathodes in a three‐electrode configuration under SPR excitation using visible light (λ>420 nm). (b) Linear sweep voltammetry (LSV) curves of bare GaN, Au/Al_2_O_3_/GaN, and Au/GaN photocathodes in the dark and under visible light illumination. (c) Steady‐state photocurrent densities of the three photocathodes measured at an applied bias of −0.4 V under light on/off cycles. (d) Comparison of photocurrent densities for the three samples.

As a proof‐of‐concept demonstration, we further carried out photoelectrochemical measurements to elucidate how ultrafast interfacial hot‐hole transfer as the critical initial step for water oxidation affects plasmon‐induced water‐splitting performance. Experiments were conducted in a neutral Na_2_SO_4_ solution using a three‐electrode configuration under visible light illumination (Figure 5a). In this system, Au/GaN serves as a photocathode, where plasmon‐induced hot holes are injected into the GaN substrate and subsequently transported to the counter electrode to drive water oxidation reaction. Meanwhile, the plasmonic hot electrons remaining on the Au NPs promote hydrogen evolution on the electrode surface. By contrast, insertion of a 5 nm Al_2_O_3_ interlayer effectively blocks interfacial charge transfer and serves as a well‐defined control to reveal the influence of interfacial charge injection on photoelectrochemical performance. As shown in Figure 5b, the Au/GaN photoelectrode exhibits a substantial improvement in photocurrent density compared to both bare GaN and Au/Al_2_O_3_/GaN. Additionally, a noticeable reduction in the onset potential is observed for the Au/GaN (0.57 V vs. RHE). Chronoamperometric I‐t curves under light on/off cycles demonstrate a rapid photoresponse and stable photocurrent (Figure 5c), confirming the robust and steady‐state catalytic performance driven by plasmonic hot carriers. Notably, at the applied bias of −0.4 V vs. RHE, the photocurrent density of the Au/GaN photocathode (1.91 µA/cm^2^) is nearly 14 times higher than that of the Au/Al_2_O_3_/GaN (0.17 µA/cm^2^) under identical illumination conditions (Figure 5d), demonstrating that ultrafast interfacial hole injection, rather than surface passivation or oxide‐mediated surface redox pathways as reported in Cu/Au/Al_2_O_3_/GaN systems [54], is the dominant factor governing PEC enhancement in our architecture. This is a result of efficient plasmon‐induced interfacial hole transfer that substantially improves the utilization efficiency of plasmonic hot electrons in the photocatalytic reaction. These findings demonstrate that interfacial engineering of plasmonic systems enables tracking of polarization‐modulated ultrafast hot hole dynamics and establishes a mechanistic link to steady‐state photocatalytic performance.

## 3 Conclusion

In summary, we establish a comprehensive temporal and energetic picture of plasmon‐induced interfacial hot‐hole transfer across the Au/GaN heterostructures. We demonstrate that an ultrafast nonthermal hole injection occurs within ∼49 fs, comparable to the timescale of nonthermal electron transfer [26]. This processes significantly reshapes the population distribution of low‐energy electrons near the Fermi level, ultimately modifying the dynamics of hot carrier relaxation. Under interband excitation, the injection process becomes even faster (∼22 fs), driven by the generation of deeper‐lying d‐band holes with higher energy. Polarization‐dependent measurements reveal that both hot‐carrier generation and interfacial charge transfer can be modulated by excitation field orientation. Implementation of this mechanism in a plasmon‐assisted PEC water‐splitting system results in a 14‐fold increase in hydrogen production. These mechanistic insights suggest a potential pathway for optimizing the performance of hot‐hole‐driven plasmonic devices [23] through nonthermal hot hole transfer demonstrated here, particularly at short excitation wavelengths where hot electrons injection is limited by the low population of high‐energy carriers generated via interband transitions [55].

## 4 Experimental Section

### 4.1 Sample Preparation

P‐type Mg‐doped GaN (0001) wafers were epitaxially grown on sapphire substrates using hydride vapor phase epitaxy (HVPE) (Hefei Kejing Material Technology Co., Ltd., China). The GaN films are 450 nm in thickness. The GaN coated wafers were then rinsed with acetone, ethanol, and deionized water in an ultrasonic bath for 10 min, and dried with nitrogen gas. Subsequently, the samples are mounted onto a molybdenum sample holder, and transferred to a preparation chamber of 2PPE with a base pressure of approximately 1 × 10^−10^ mbar. Samples were cleaned by several cycles of 650 eV Ar^+^ sputtering for 10 min, each followed by annealing at 600°C for 20 min. This produces a long‐range order surface as confirmed by low‐energy electron diffraction. For Au film deposition, the GaN wafer was transferred under vacuum condition to an electron‐beam evaporation system in an UHV environment with a base pressure of 1 × 10^−9^ mbar. Subsequently, a 1 nm gold thin film was deposited onto the GaN surface at a deposition rate of 1 Å s^−1^, followed by annealing at 450°C for 2 h in UHV to form gold nanoparticles and to achieve good contact with the underlying GaN facilitating charge transfer. The thickness of the deposited Au film was monitored by a quartz crystal microbalance (QCM) sensor. For the Au/Al_2_O_3_/GaN sample, the preparation process was same as the aforementioned processes, except that a 5‐nm‐thick Al_2_O_3_ layer was deposited on GaN surface using atomic layer deposition (ALD) prior to the gold film deposition. The deposition of Al_2_O_3_ thin films were conducted using a custom‐designed hot‐wall stainless‐steel ALD reactor. The system, equipped with a turbomolecular pump backed by a roughing pump, achieved a base pressure below 2 × 10^−7^ mbar. Trimethylaluminum (98%, Strem Chemicals, Inc.) and H_2_O served as the aluminum and oxygen precursors, respectively, both maintained at room temperature. The precursors were alternately introduced via ALD diaphragm valves using a vapor‐draw method without carrier gas. The reactor walls were heated to 125°C, while the tubing and fittings remained unheated. The substrate temperature was maintained at 170°C throughout the deposition. Each precursor exposure was followed by a pump/purge/pump sequence to maintain the base pressure at 3–5 × 10^−6^ mbar. This sequence consisted of 15 s of pumping, a 100 ms Ar purge, and another 15 s of pumping. The growth rate of Al_2_O_3_ was determined to be 0.1 nm per cycle.

### 4.2 Time‐Resolved Two‐Photon Photoemission Spectroscopy (tr‐2PPE)

To track the occupation dynamics of surface electronic states and photoexcited electrons, time‐resolved two‐photon photoemission spectroscopy is employed. All tr‐2PPE measurements are performed under UHV, at a base pressure of ca. 1.0 × 10^−10 ^mbar at room temperature (20.6°C). tr‐2PPE requires ultrafast pump and probe pulses, which are generated from a Ti:Sa laser in our set‐up. The laser pulses of a Coherent Vitara Titanium Sapphire (Ti:Sa) oscillator are passed through a grating‐based compressor/expander unit, expanded, and then amplified by a Coherent RegA 9050 amplifier pumped by a Verdi 12 laser. This results in pulses at 800 nm and a full width at half maximum (FWHM) of ca. 50 fs, with pulse energies of 10 µJ. To perform tr‐2PPE, electrons in the sample are photoexcited by a pump pulse, undergo ultrafast relaxation processes characteristic to the structure being studied, and after an adjustable time delay are emitted by a probe pulse; this forms the two‐photon photoemission process. To allow for the photon energies of these pump and probe pulses to be adjusted, the amplified 800 nm Ti:Sa laser is passed through beamsplitters, with the remaining (majority) of the light frequency doubled to 400 nm using second harmonic generation in a β‐barium borate (BBO) crystal. This 400 nm light pumps two non‐collinear parametric amplifiers (NOPA), with the white light continuum provided by hard‐focusing the remaining 800 nm light through sapphire disks. One of the NOPAs generates the visible (VIS) pump beam used in tr‐2PPE, with its photon energies tunable from 470 nm (2.64 eV) to 560 nm (2.21 eV), and 80 nJ pulse energy immediately behind the NOPA. The other NOPA outputs pulses tuned to 545 nm with approximately 220 nJ pulse energy. This is then frequency doubled, with the resulting pulse serving as the UV probe pulse in tr‐2PPE measurements. The UV probe pulse used in this study is 276 nm. Both the pump and probe pulses are individually chirp‐compensated using a pair of quartz prisms, achieving a measured FWHM of approximately 40 fs at the sample. The measurement spot size on the sample is determined by the overlap of the VIS and UV beam spots of around 100 × 100 µm^2^. VIS pump pulse energies were controlled by neutral density filters. Photoemitted electrons are detected using a time‐of‐flight (TOF) detector placed ca. 3–5 mm from the sample surface to optimize angular acceptance while minimizing the detection of secondary (scattered) electrons. The TOF detector has a 7.3° acceptance angle and an energy resolution of approximately 50 meV, and the pump and probe beams are incident at ca. 45° relative to the surface normal. Measurements of cross‐correlation (CC, VIS + UV) were carried out on a Cu (100) single crystal surface, which features an occupied surface state. This well‐characterized reference surface exhibits emission via virtual intermediate states in the sp‐bandgap under the photon energies used. Prior to the CC measurements, the Cu surface underwent a preparation process involving three cycles of sputtering (Ar^+^, 650 eV, 1.5 × 10^−5^ mbar), followed by annealing at 527°C for 20 min after each cycle.

### 4.3 Surface Photovoltage Microscopy (SPVM)

SPV images were acquired using a commercial atomic force microscope (AFM) system (Bruker Dimension Icon V) in light‐modulated Kelvin probe force microscopy (KPFM) mode under ambient conditions. Amplitude‐modulated KPFM in lift mode was employed to probe the surface potential of the samples. To ensure high spatial resolution and minimize crosstalk artifacts, the AFM tip scanning rate was set to 0.5 Hz, with a lift height maintained at 10 nm. Platinum/iridium‐coated silicon probes (SCM‐PIT) were used, with a nominal spring constant of 3 N/m, a tip radius of approximately 25 nm, and a resonance frequency of around 75 kHz. The recorded surface potential corresponds to the work function difference between the probe tip and the sample surface. For light‐modulated KPFM, a monochromatic light source (550 nm, 5 mW/cm^2^), generated by a Xenon lamp with a Zolix Omni‐λ 500 monochromator, was focused on the sample surface to excite the Au NPs. SPV maps were obtained by subtracting the surface potential image under dark conditions from the illuminated surface potential image, reflecting the spatial distribution of photogenerated charges across the sample surface.

### 4.4 Ultraviolet‐Visible (UV‐vis) Absorption Spectroscopy

To characterize the optical absorption properties of the samples, UV‐vis absorption spectroscopy is measured using a PerkinElmer Lambda 950 spectrophotometer. This enables high accuracy across a broad spectral range from 190 to 3300 nm due to a dual‐beam optical system and a high‐performance photomultiplier tube (PMT) for the UV‐Vis range, complemented by an indium gallium arsenide (InGaAs) detector for the NIR range.

### 4.5 X‐Ray Photoelectron Spectroscopy (XPS)

XPS measurements were carried out at room temperature utilizing a SPECS Focus 500 monochromator paired with a Phoibos 100 electron energy analyzer. The system employed an aluminum K‐alpha X‐ray source (1486.74 eV) operated at 200–300 W. The experiments were performed with a photoelectron take‐off angle of 90°, an angle of 54.7° between the X‐ray source and the analyzer, and a pass energy set to 10 eV. For high‐resolution core‐level peaks, data acquisition was conducted with 0.05 eV energy steps, followed by post‐processing using Shirley background subtraction. To measure XPS under illumination conditions, a 532 nm (∼100 mW/cm^2^) laser was focused onto the sample surface to excite the sample.

### 4.6 Ultraviolet Photoelectron Spectroscopy (UPS)

UPS was employed to determine the surface work functions and to characterize the valence band structure of the samples. All measurements were conducted in a UHV chamber with a base pressure ∼10^−10^ mbar at room temperature using the same instrumentation as the XPS analysis. Helium I (He‐I) radiation with a photon energy of 21.2 eV was utilized as the UV light source, with the discharge lamp operating at 25–35 W. The incident radiation was directed at an angle of 50° relative to the normal surface. UPS was also measured at a bias potential of −5.0 V between the sample and the detector to accurately define the secondary electron cutoff.

### 4.7 Photoelectrochemical Measurement

The photoelectrochemical performances were measured by the electrochemical workstation in a typical three‐electrode cell. The prepared electrodes, Pt foil, and Ag/AgCl were used as the working electrode, counter electrode, and reference electrode, respectively. 0.5 m Na_2_SO_4_ aqueous solution was used as the electrolyte. A Xe lamp (300 W cm^−2^) with a long‐pass band filter (>420 nm) was used as the light source. Photocurrent density versus voltage curves were recorded from −0.8 to 0.8 V versus RHE with a scan rate of 10 mV s^−1^.

## Funding

The authors are thankful for the financial support by the National Key R&D Program of China (grant nos. 2024YFA1210802 and 2021YFA1500600), the Fundamental Research Center of Artificial Photosynthesis (FReCAP), the National Natural Science Foundation of China (22325205, 22088102 and 22472170), CAS Projects for Young Scientists in Basic Research (YSBR‐004), Fundamental Research Funds for the Central Universities, 20720220011, New Cornerstone Science Foundation through the XPLORER PRIZE, and German Research Foundation (DFG project PAK 981, project no. FR 4025/2‐1, KR4816/1‐1). Y.G. acknowledges the financial support from Dalian Institute of Chemical Physics, CAS, China.

## Conflicts of Interest

The authors declare no conflicts of interest.

## Supporting information




**Supporting File**: advs75037‐sup‐0001‐SuppMat.docx.

## Data Availability

The data that support the findings of this study are available from the corresponding author upon reasonable request.
