# Supplementary material for: Energy‐Resolved Femtosecond Dynamics of Plasmon‐Induced Hole Injection at Au/GaN Heterointerfaces
Source: Adv Sci (Weinh). 2026 Mar 30;13(33):e23801. doi: 10.1002/advs.202523801 (PMC13271609; doi:10.1002/advs.202523801)
Supplement: Supplementary file 1 — Supporting File: advs75037‐sup‐0001‐SuppMat.docx. [file ADVS-13-e23801-s001.docx]

**Supporting Information**

**Energy-resolved Femtosecond Dynamics of Plasmon-induced Hole Injection at Au/GaN Heterointerfaces**

Yuying Gao^*^, Yuxin Xie, Jonathan Diederich, Christian Höhn, Klaus Schwarzburg, Fengtao Fan, Can Li, Roel van de Krol, and Dennis Friedrich^*^

Y. Gao, J. Diederich, C. Höhn, K. Schwarzburg, R. van de Krol, D. Friedrich

Institute for Solar Fuels, Helmholtz-Zentrum Berlin für Materialien und Energie GmbH, Berlin 14109, Germany

E-mail: yuying.gao@helmholtz-berlin.de; friedrich@helmholtz-berlin.de

Y. Gao, Y. Xie, F. Fan, C. Li
State Key Laboratory of Catalysis, Dalian National Laboratory for Clean Energy, Dalian Institute of Chemical Physics, Chinese Academy of Sciences, Dalian 116023, China

E-mail: yuying.gao@helmholtz-berlin.de; friedrich@helmholtz-berlin.de

J. Diederich, R. van de Krol
Institut für Chemie, Technische Universität Berlin, Berlin 10623, Germany

**Supporting Note S1: Calculation of plasmon dephasing time**

The plasmon dephasing time ($\tau$) of Au NPs was determined using the following equation^[1, 2]^:

$\tau=\frac{2\text{ħ}}{w}$ (1)

where $w$is the full width at half maximum (FWHM) of SPR absorption band. Experimental results indicate the values of $w$ for Au/GaN and Au/Al_2_O_3_/GaN of 0.64 eV and 1.10 eV, respectively. The plasmon dephasing times are calculated to be 0.91 fs for Au/GaN and 1.56 fs for Au/Al_2_O_3_/GaN. These timescales are significantly shorter than the rise time of the photoelectron signals measured by tr-2PPE, and the difference between the two systems is less than 1 fs. Therefore, we conclude that the plasmon dephasing time has a negligible effect on the observed rise time of the photoemission signal across different systems.

To further clarify the origin of the observed rise-time differences, we also considered the possible influence of nanoparticle size dispersion and variations in optical absorption strength. The Au NPs in this study are ultrasmall, with an average height of ~2.2 nm and a standard deviation of 1.2 nm, as confirmed by AFM (Figure 1a, Figure S3). While size dispersion can influence the SPR linewidth via enhanced surface scattering and minor inhomogeneous broadening, AFM confirms that Au/GaN and Au/Al_2_O_3_/GaN samples have nearly identical particle density and size distribution. Therefore, differences in ultrafast dynamics primarily reflect interfacial charge transfer rather than morphological variability.

Importantly, tr-2PPE measurements probe electrons from individual nanoparticles, such that the measured rise times and lifetimes reflect intrinsic carrier dynamics rather than ensemble-averaged optical responses. Due to the surface-specific nature of photoemission and the high degree of carrier localization, the extracted rise times and lifetimes are fundamentally dictated by the electronic band structure and interfacial potentials. This is further corroborated by fluence-dependent measurements (Figures S12-S13), which demonstrate that the carrier dynamics are independent of excitation power. Consequently, minor variations in particle size mainly affect the SPR absorption amplitude but do not alter the temporal evolution of hot electrons and holes, supporting the robustness of the mechanistic conclusions presented in the main text.

**Supporting Note S2: Fitting procedure of photoelectron dynamics**

The experimentally measured dynamics were fitted to determine the lifetime of hot electrons. For high-energy hot electrons (>1.2 eV), the energy-resolved photoelectron intensity dynamics were fitted using a Gaussian distribution convoluted with an exponential function, as provided below^[3]^:

$I\left( t \right)$ $=A\cdot e^{\left( -\frac{\left( t-t_{0} \right)^{2}}{2w^{2}} \right)*}e^{-\frac{t}{\tau}}$ (2)

where the Gaussian function accounts for the cross-correlation (CC) of pump and probe pulses, as shown in Figure 3e. The exponential term captures the signal decay over time, with a characteristic time 𝜏.

For low-energy electrons (≤1.2 eV), the energy-resolved photoelectron dynamics were fitted with the following function:

$\frac{dI\left( t \right)}{dt}=A\cdot e^{\left( -\frac{\left( t-t_{0} \right)^{2}}{2w^{2}} \right)}-\frac{I\left( t \right)}{\tau}+B\cdot\left( 1-e^{-\frac{t}{\tau_{r}}} \right)$ (3)

The first term represents CC profiles, the second term accounts for the relaxation of excited low-energy electrons with time constant $\tau$, and the third term describes the decrease in the photoelectron intensity caused by the thermalization of high-energy electrons and the plasmon-induced hole injection processes.

To determine the timescale of the ultrafast interfacial hole transfer, the rise time ($\tau_{r}$) of low-energy electron (0.5 eV vs E_F_) was analysed for both samples, as low-energy electrons distributed near the Fermi level are easily affected by the hot hole injection processes. For the Au/Al_2_O_3_/GaN sample, the rise time ($\tau_{r}$) of low-energy electrons is governed by the hot electron generation rate ($\tau_{gene}$) and the thermalization of high-energy electrons ($\tau_{ther}$), while for the Au/GaN sample, $\tau_{r}$is additionally influenced by charge transfer. Thus, the rise time for Au/Al_2_O_3_/GaN sample can be described using the following equation:

$\tau_{r}^{-1}=\tau_{gene}^{-1}+\tau_{ther}^{-1}$ (4)

To account for the different relaxation time of high-energy electrons in tr-2PPE for the samples, the rise time for Au/GaN sample is given by:

$\tau_{r}^{-1}=\tau_{gene}^{-1}+\tau_{ther*}^{-1}+\tau_{transfer}^{-1}$ (5)

where time constant $\tau_{transfer}$ is associated with hole transfer process. The thermalization time constant of high-energy electrons can be obtained from the fitting of kinetic trace using the equations above. Assuming that the generation rates of electrons at different energy levels are identical, this is related to the intrinsic properties of the metal. The hole transfer time scale is determined to be 49±14 fs; the error is the standard deviation of fitted electron lifetimes.


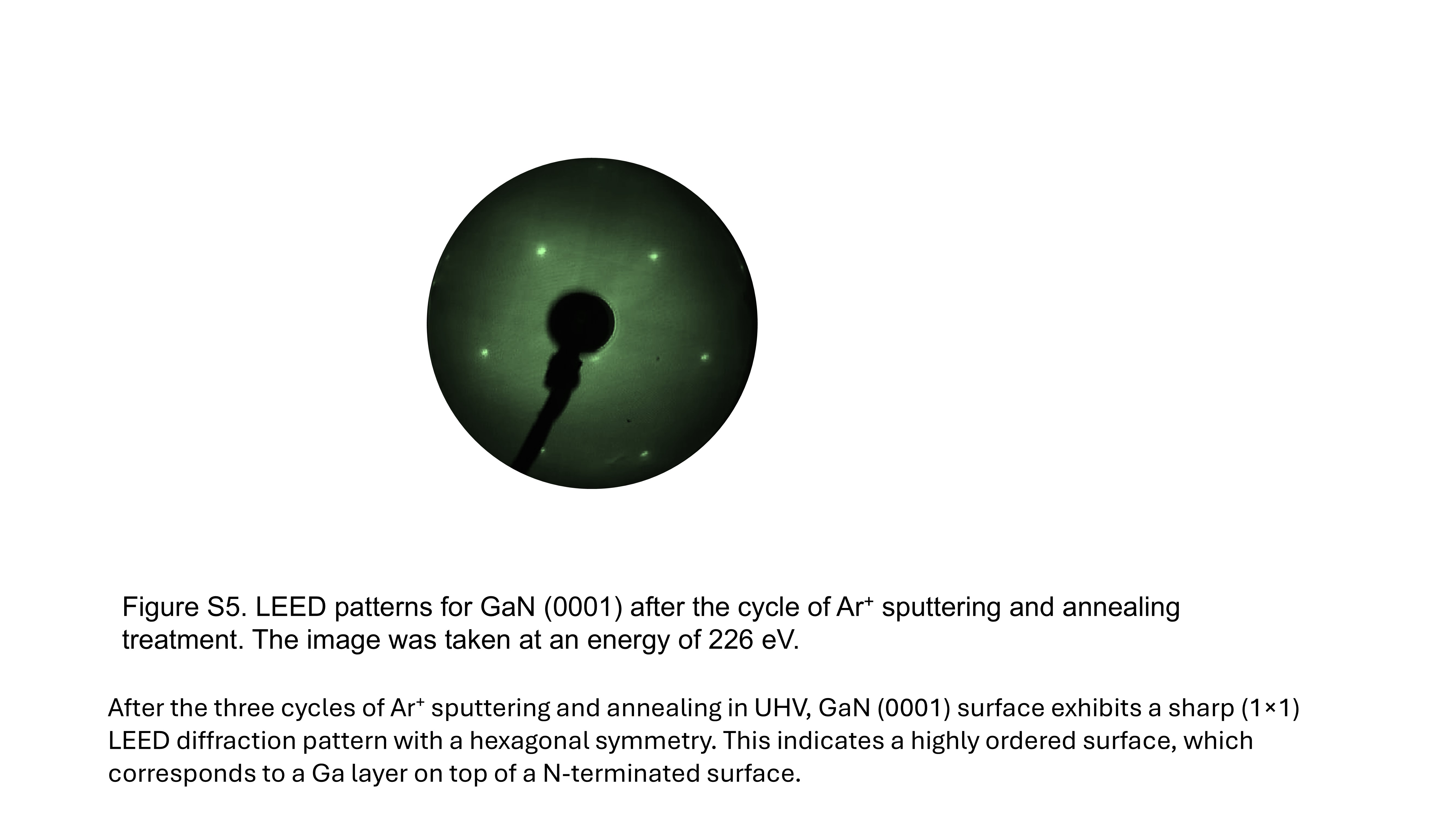


**Figure S1.** LEED patterns for GaN (0001) after three cycles of Ar^+^ sputtering and annealing treatment. The image was taken at an energy of 226 eV.

After three cycles of Ar^+^ sputtering and annealing in UHV (see Methods for details), the GaN (0001) surface exhibits a sharp (1×1) LEED diffraction pattern with hexagonal symmetry. This indicates a highly ordered surface, which corresponds to a Ga layer on top of a Ga-terminated surface.^[4]^


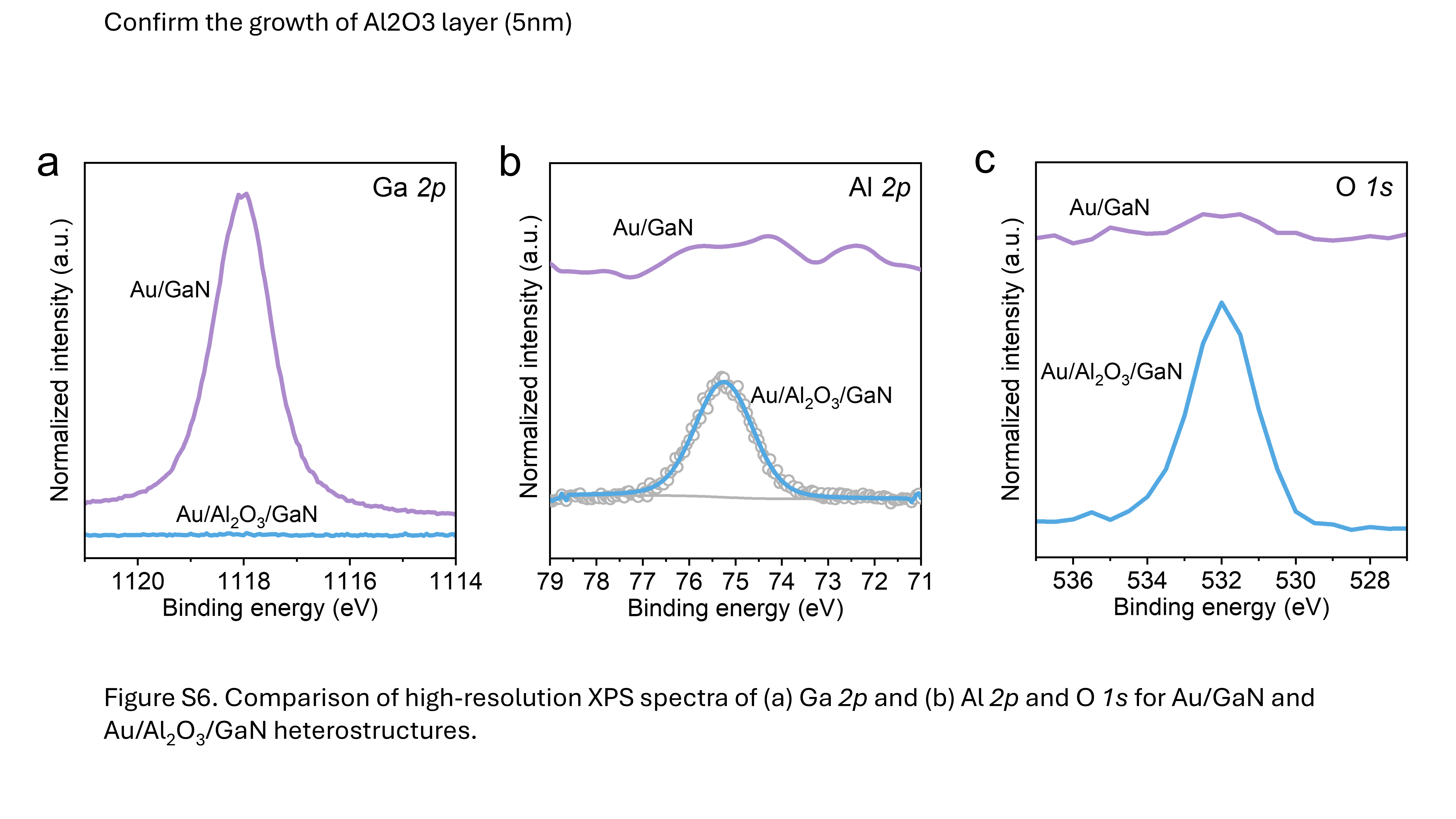


**Figure S2.** Comparison of high-resolution XPS of (a) Ga 2*p*, (b) Al 2*p*, and (c) O 1*s* for Au/GaN and Au/Al_2_O_3_/GaN heterostructures. Due to the low electron IMFP of around 2 nm for the 1486.74 eV excitation used,^[5]^ no clear Ga signal is observed from the Au/GaN surface after inserting a 5 nm Al_2_O_3_ interlayer. The Al 2*p* peak verifies the presence of the Al_2_O_3_ layer. Similarly, the O 1*s* peak in c is only observed for the Au/ Al_2_O_3_/GaN sample.


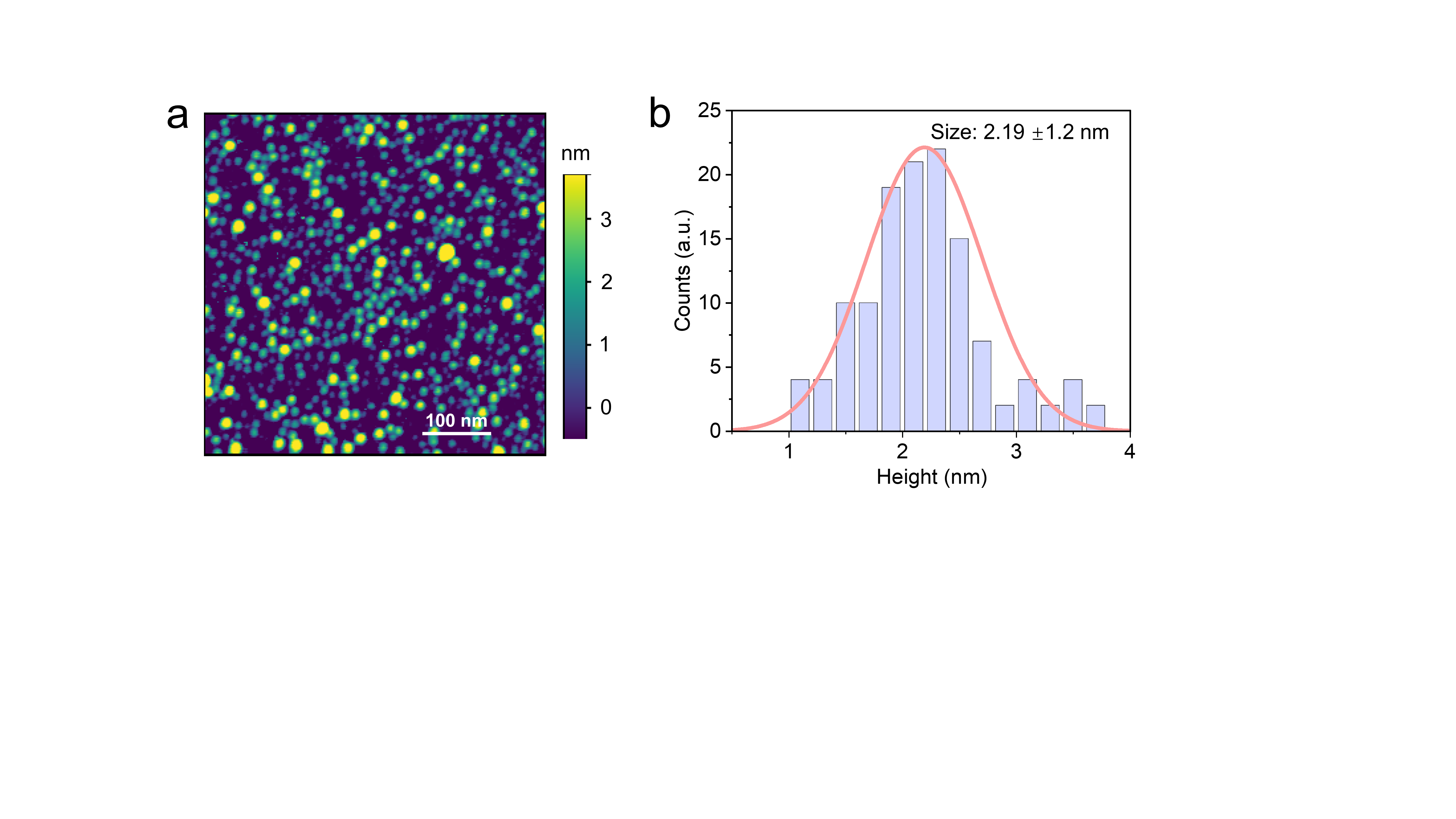


**Figure S3.** (a) AFM image of Au nanoparticles on the Al_2_O_3_/GaN substrate. (b) Height distributions of Au nanoparticles and the Gaussian fits. The average height of Au nanoparticles is 2.19 nm.


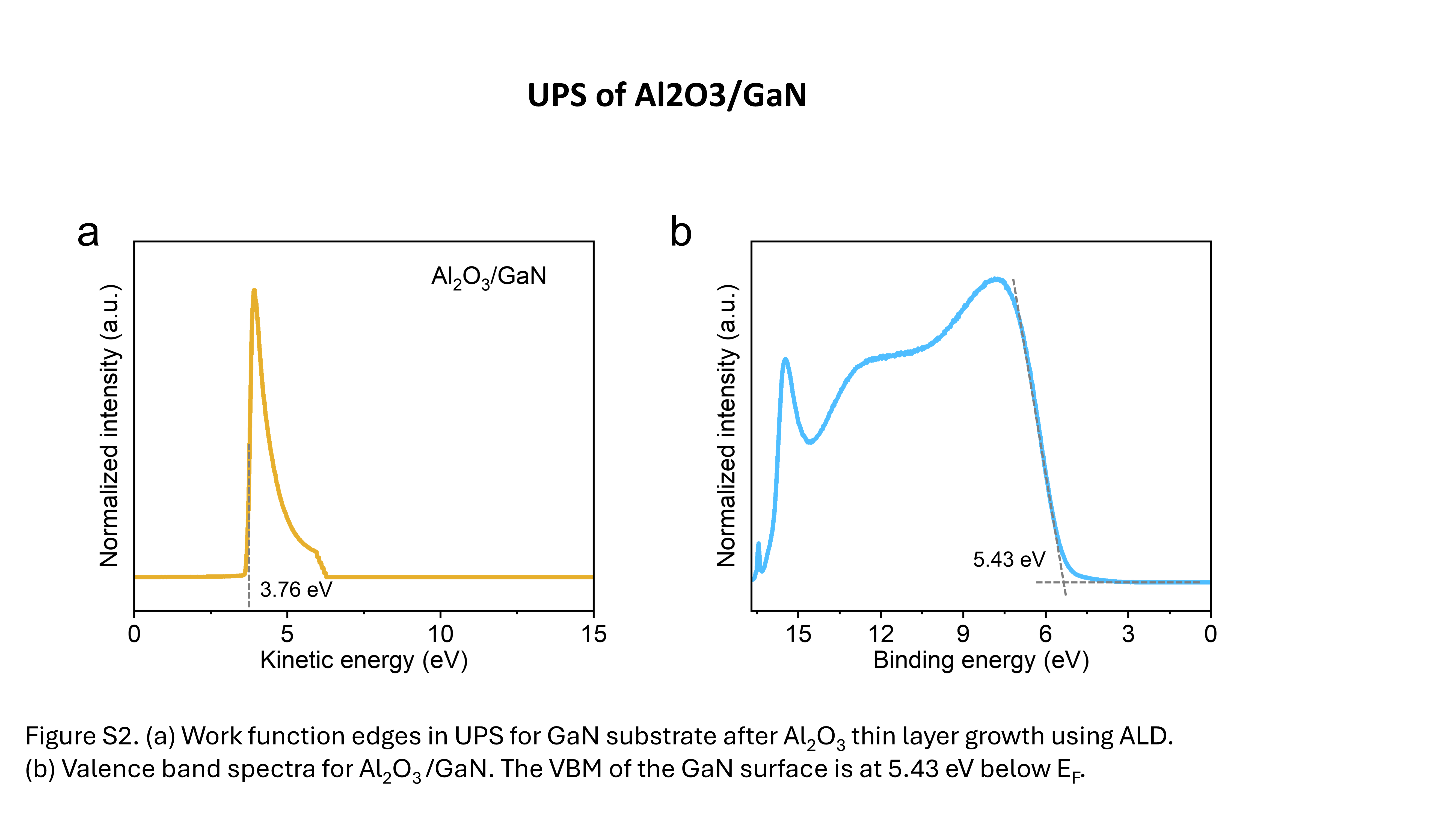


**Figure S4.** (a) Work function edges in UPS for the GaN substrate after Al_2_O_3_ thin layer growth vis ALD. (b) Valence band spectra for Al_2_O_3_ /GaN. The VBM of the Al_2_O_3_ surface is found 5.43 eV below E_F_.


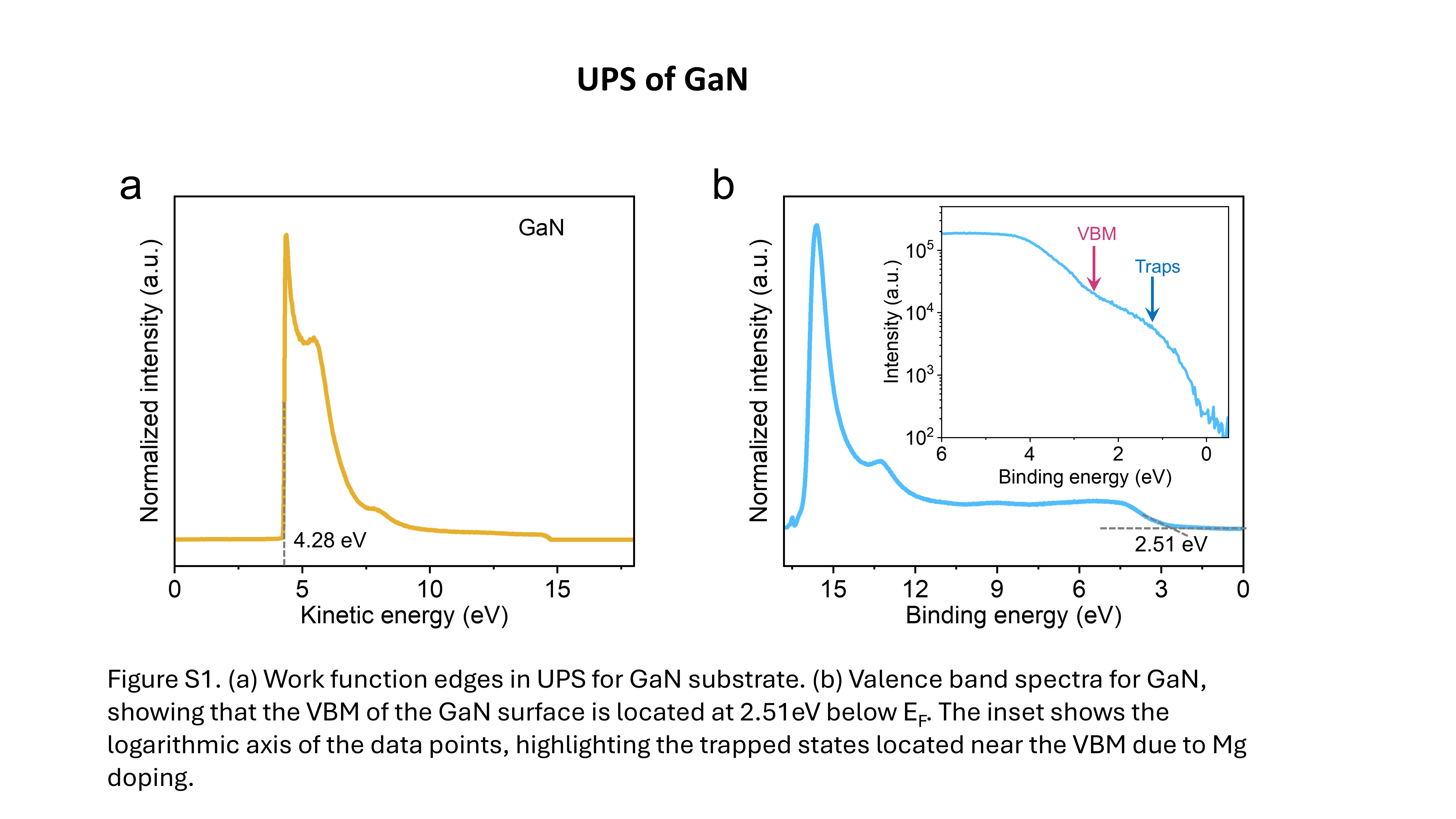


**Figure S5.** (a) Work function edges in UPS for the bare GaN substrate. (b) Valence band spectra for GaN, showing that the VBM of the GaN surface is located at 2.51eV below E_F_. The inset shows data on a logarithmic axis, highlighting trapped states located near the VBM due to Mg doping.


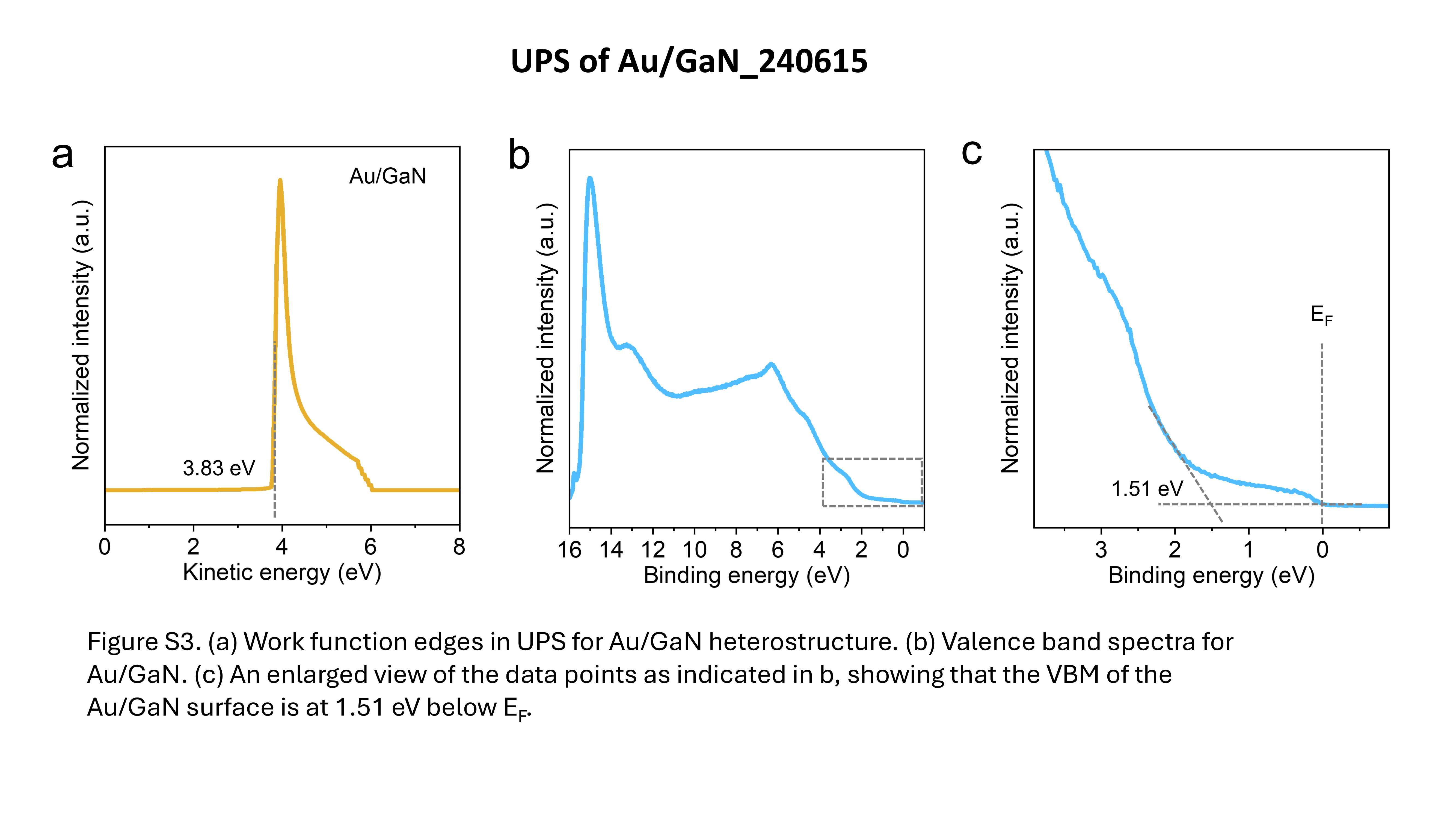


**Figure S6.** (a) Work function edge in UPS for the Au/GaN heterostructure at 3.83 eV vs vacuum. (b) Valence band spectrum for Au/GaN, with an enlarged view of the data points in (c), showing the VBM at 1.51 eV below E_F_.


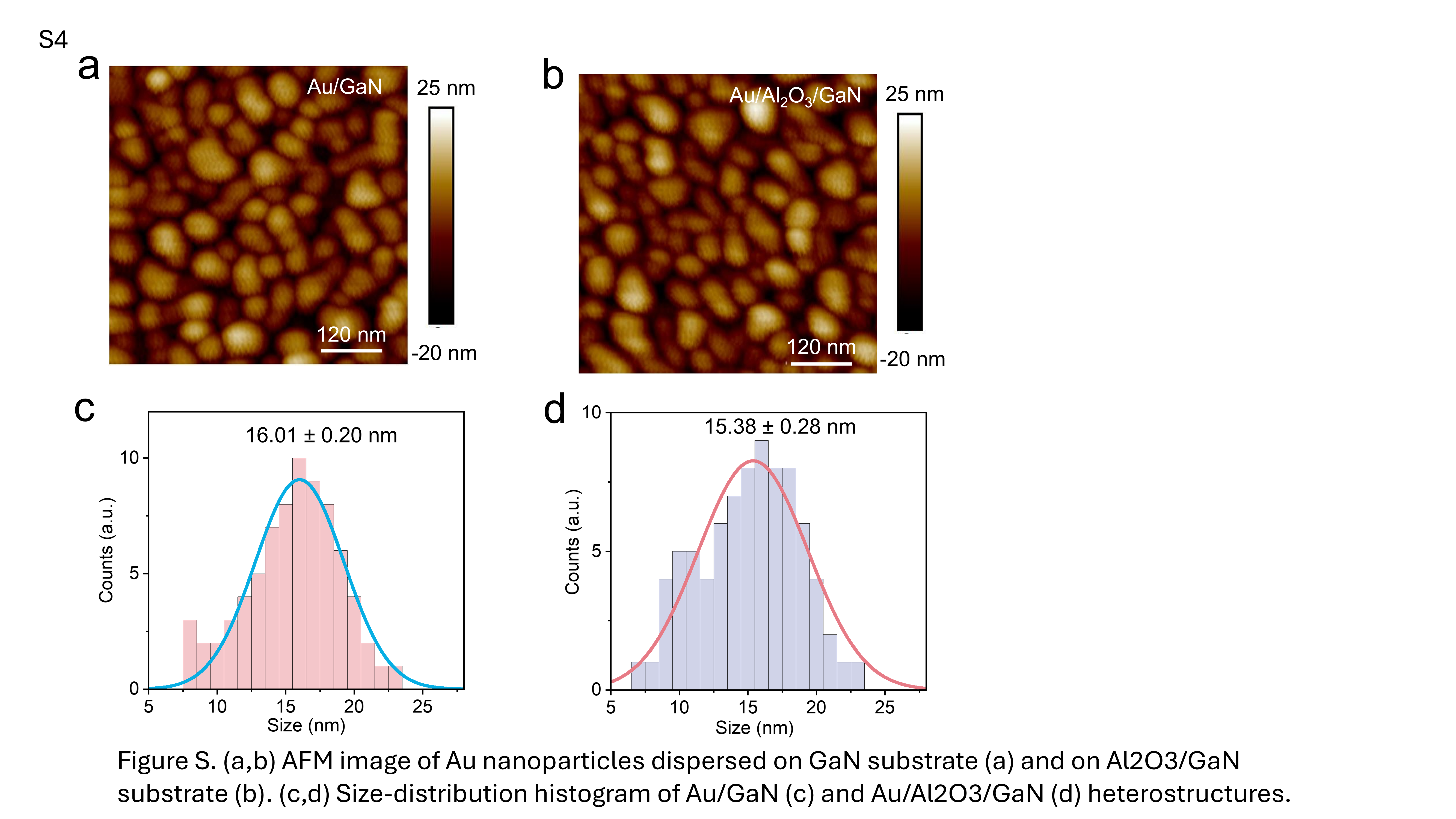


**Figure S7.** (a,b) AFM image of Au nanoparticles dispersed on GaN substrate (a) and on Al_2_O_3_/GaN substrate (b). (c,d) Size-distribution histogram of Au nanoparticles for Au/GaN (c) and Au/Al_2_O_3_/GaN (d) heterostructures.


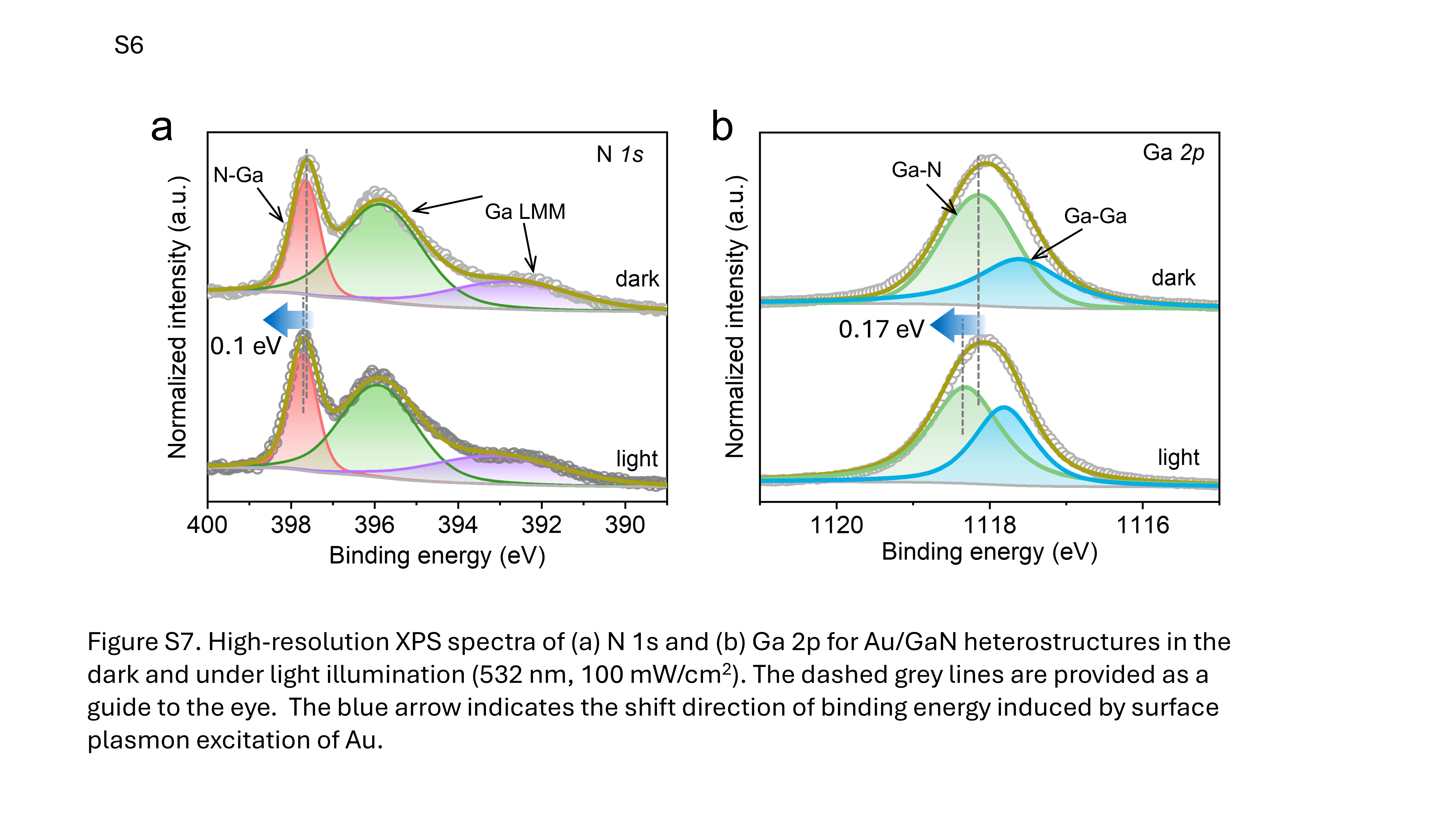


**Figure S8.** High-resolution XPS spectra of (a) N 1*s* and (b) Ga 2*p* for Au/GaN heterostructures in the dark and under light illumination (532 nm, 100 mW/cm^2^). The dashed grey lines are included as guides to the eye, denoting the N–Ga and Ga–N bond peaks and their respective shifts upon illumination. The blue arrow indicates the shift in binding energy induced by surface plasmon excitation of Au. The increased binding energy of the peaks corresponds to an increase in positive charge in the GaN substrate under illumination, indicating hole-transfer from the Au NPs into the substrate, or electron transfer in the opposite direction.


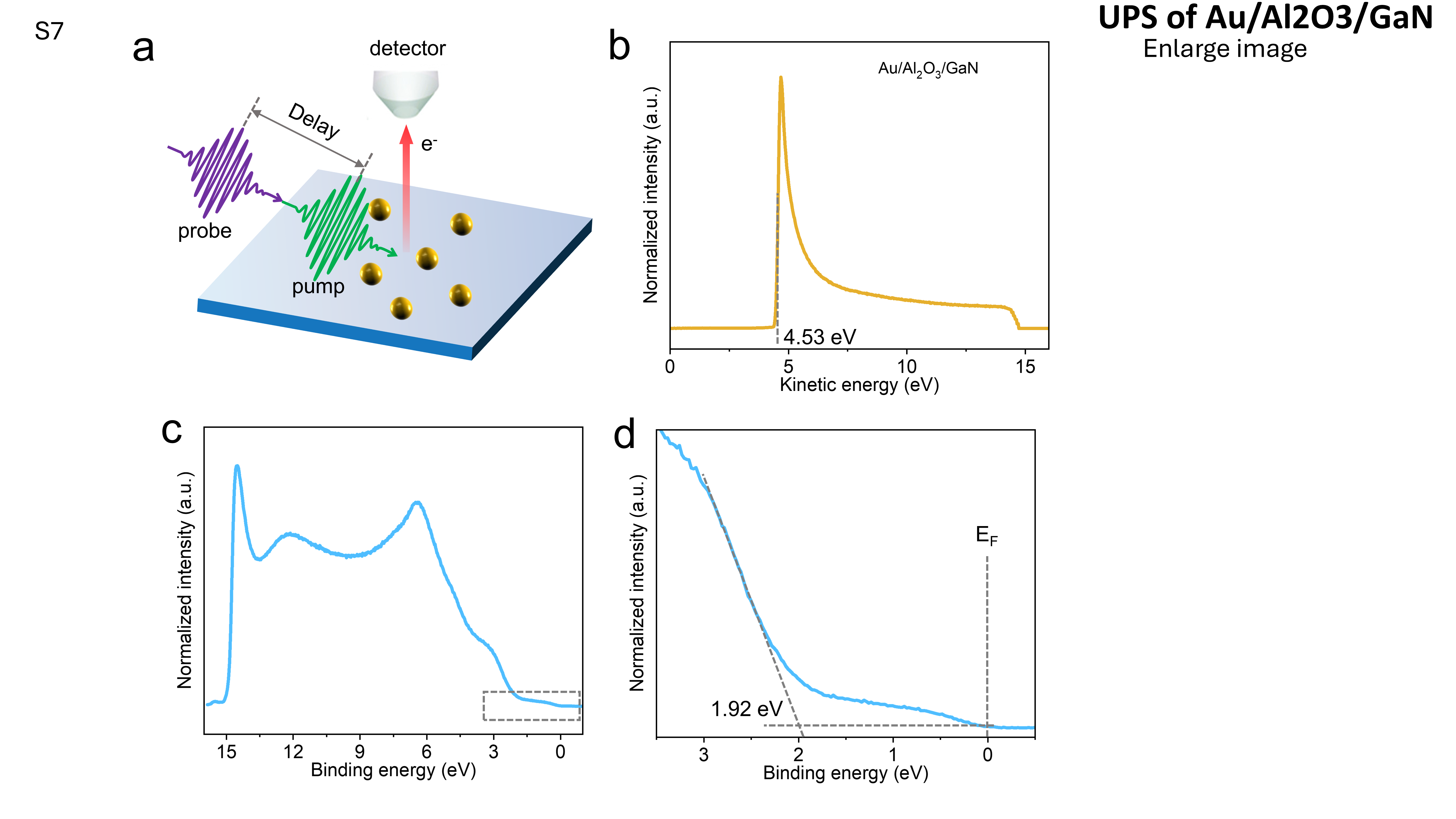


**Figure S9.** (a) Schematic of the pump-probe configuration and photoelectron detection using a time-of-flight (TOF) detector in tr-2PPE measurement. (b) Work function edges in UPS for Au/Al_2_O_3_/GaN sample. (c) Valence band spectra for Au/Al_2_O_3_/GaN. (d) An enlarged view of the data points as indicated in c is given, showing that the VBM of the Au/Al_2_O_3_/GaN surface is located at 1.92 eV below E_F_.


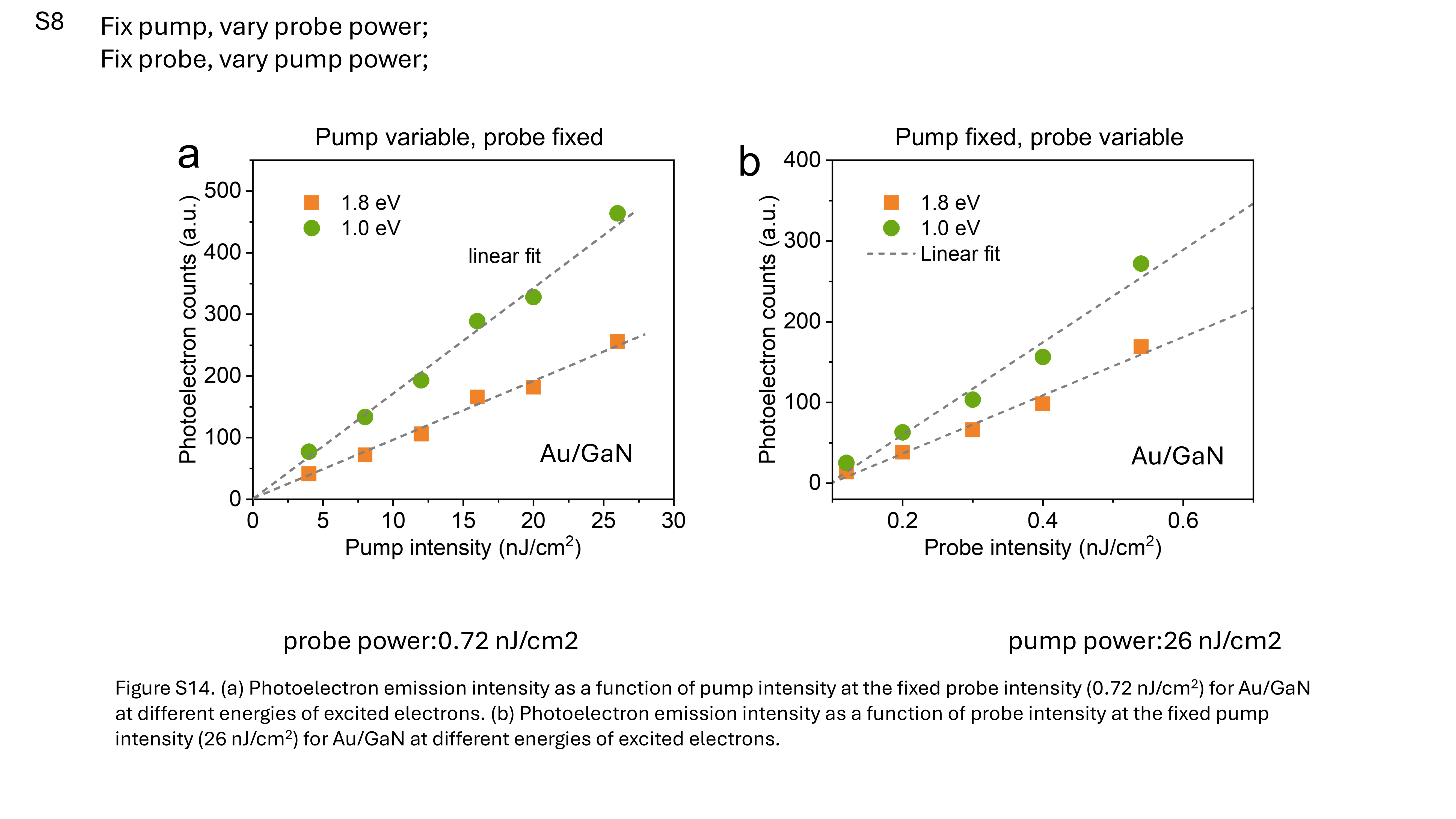


**Figure S10.** (a) Photoelectron emission intensity as a function of pump intensity at fixed probe intensity (0.72 nJ/cm^2^) for Au/GaN at different excited electron energies. (b) Photoelectron emission intensity as a function of probe intensity at fixed pump intensity (26 nJ/cm^2^) for Au/GaN at different excited electron energies.


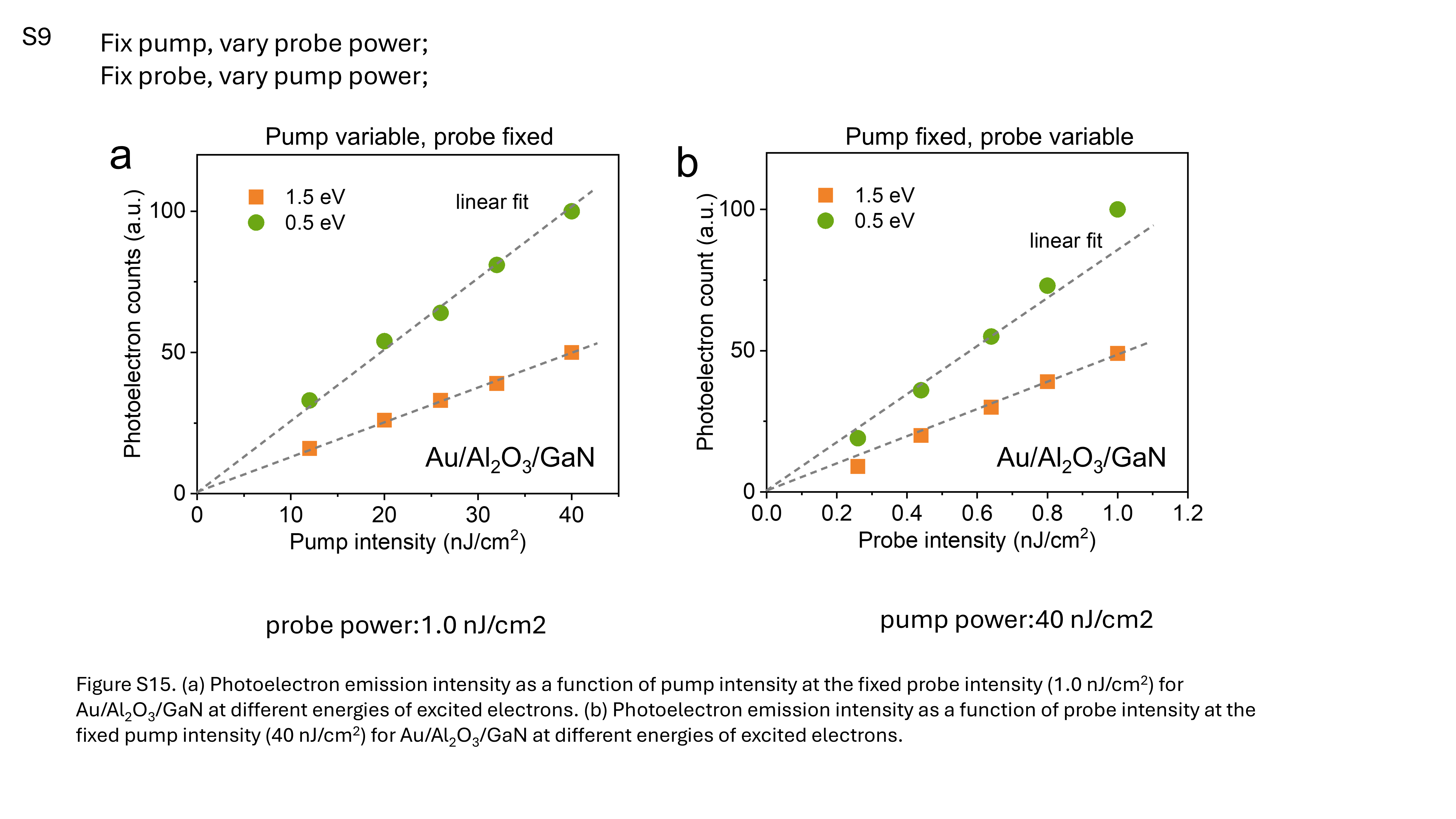


**Figure S11.** (a) Photoelectron emission intensity as a function of pump intensity at the fixed probe intensity (1.0 nJ/cm^2^) for Au/Al_2_O_3_/GaN at different energies of excited electrons. (b) Photoelectron emission intensity as a function of probe intensity at fixed pump intensity (40 nJ/cm^2^) for Au/Al_2_O_3_/GaN at different energies of excited electrons.


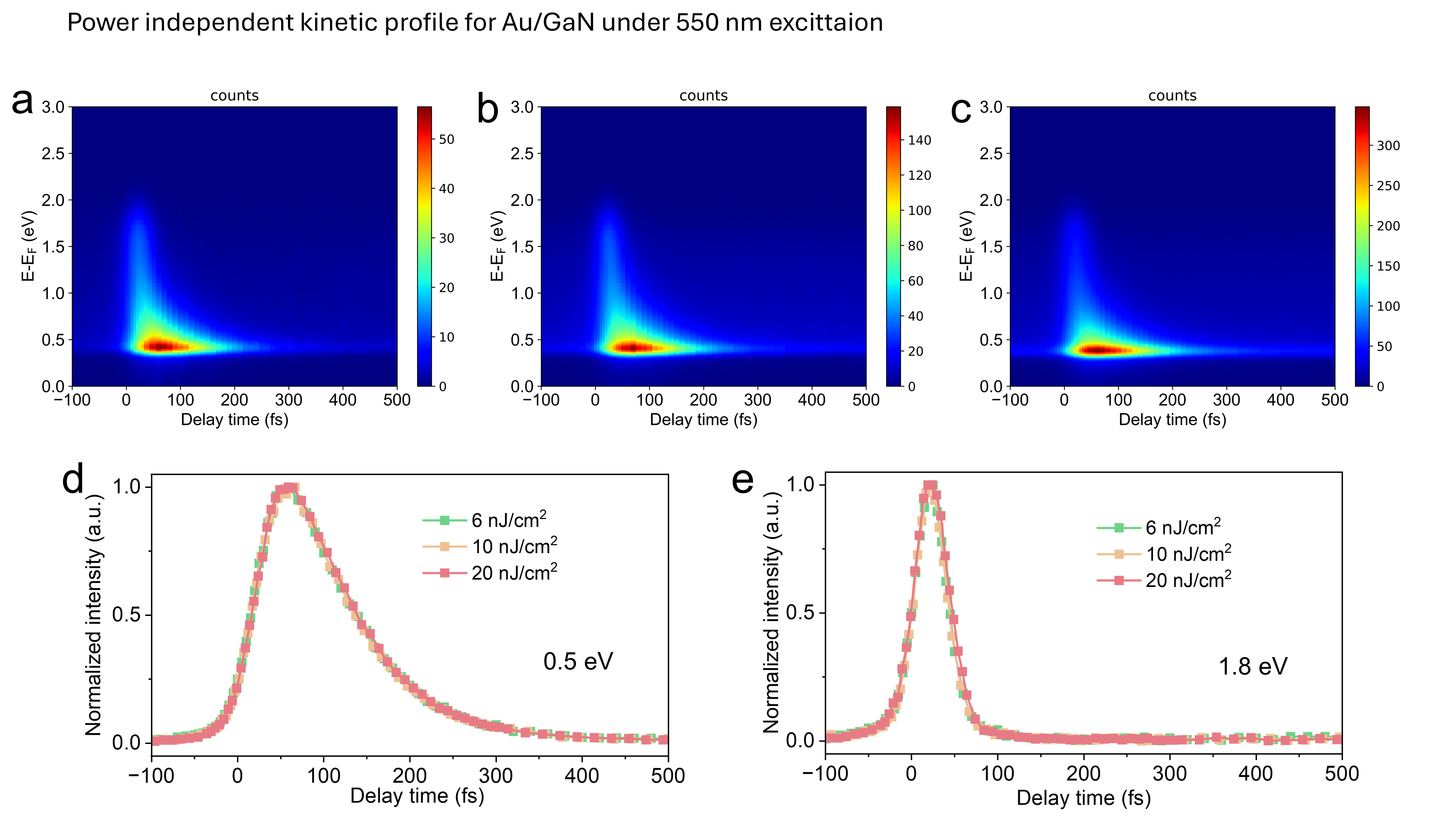


**Figure S12.** (a-c) Representative pseudo-color tr-2PPE spectra as a function of pump-probe delay time for Au/GaN at a pump flux of (a) 6 nJ/cm^2^, (b) 10 nJ/cm^2^ and (c) 20 nJ/cm^2^. (d,e) Pump power dependence of hot electron dynamics for Au/GaN at a (d) low energy of 0.5 eV and (e) high energy of 1.8 eV relative to E_F_.


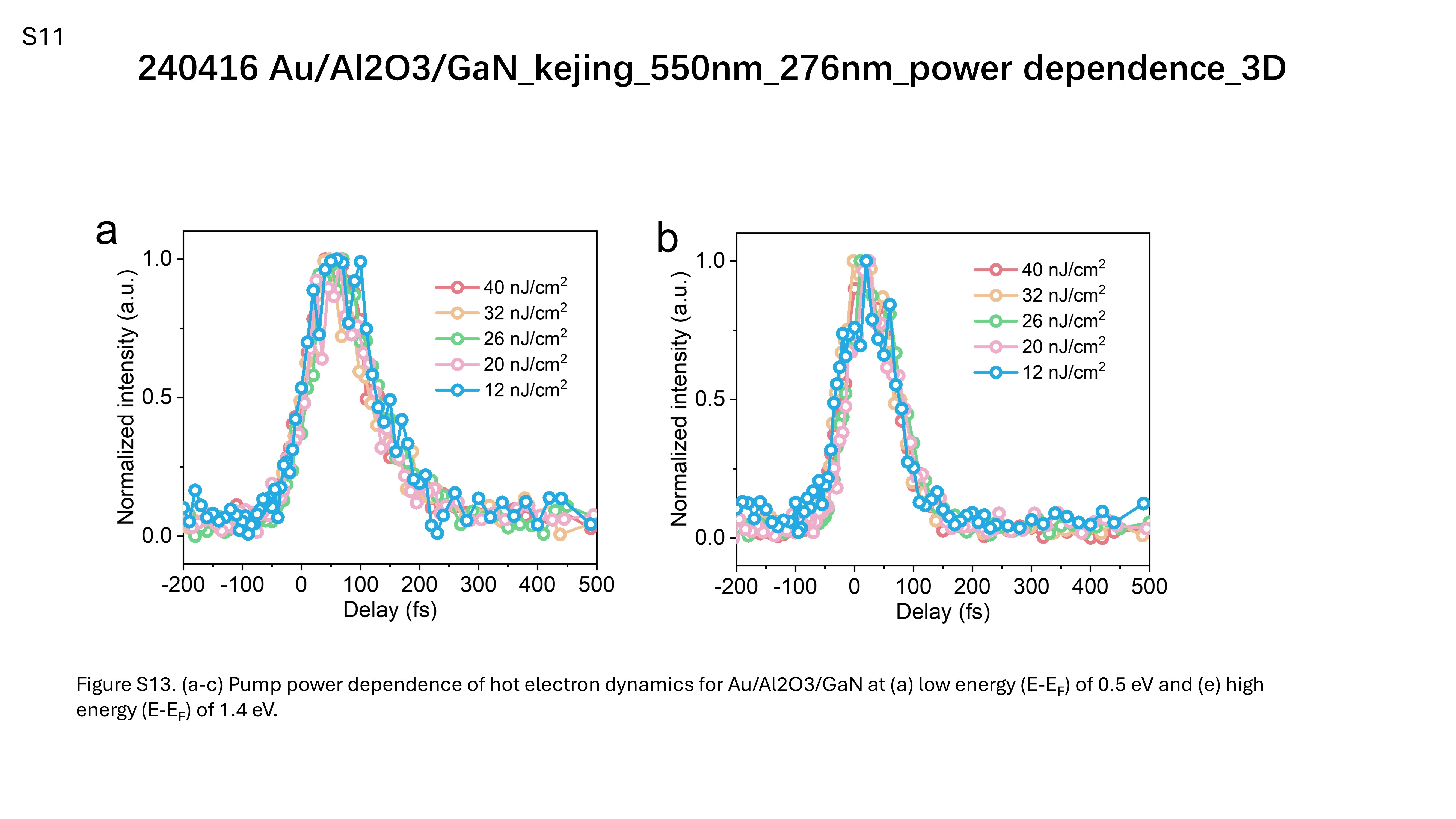


**Figure S13.** (a-c) Pump power dependence of hot electron dynamics for Au/Al_2_O_3_/GaN at a (a) low energy (E-E_F_) of 0.5 eV and (b) high energy (E-E_F_) of 1.4 eV, measured at a pump energy of 550 nm and probe energy of 276 nm.


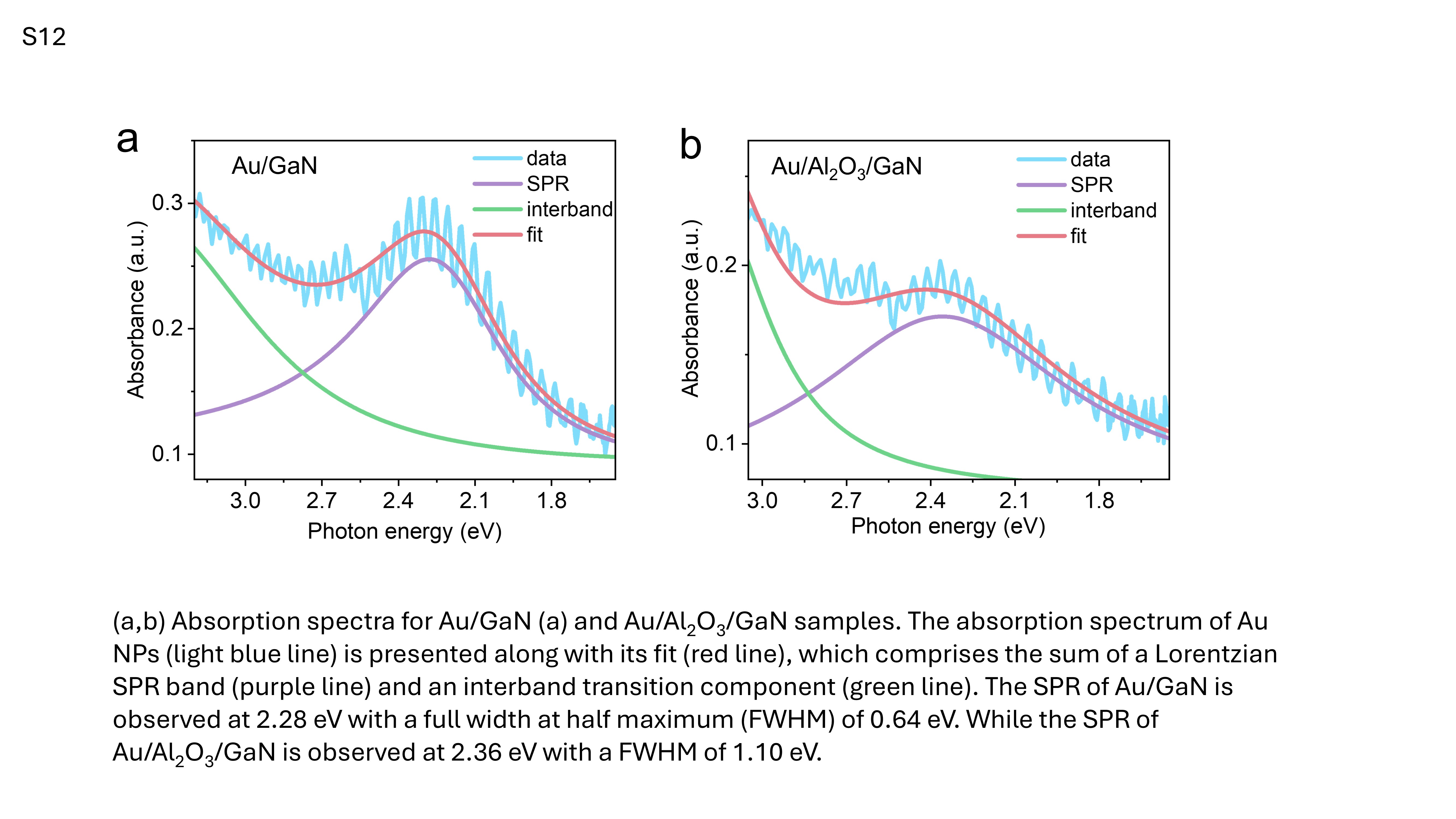


**Figure S14.** (a,b) Absorption spectra for Au/GaN (a) and Au/Al_2_O_3_/GaN samples. The absorption spectrum of Au NPs (light blue line) is presented along with its fit (red line), which comprises the sum of a Lorentzian SPR band (purple line) and an interband transition component (green line). The SPR of Au/GaN is observed at 2.28 eV with a full width at half maximum (FWHM) of 0.64 eV, while the SPR of Au/Al_2_O_3_/GaN is observed at 2.36 eV with a FWHM of 1.10 eV.


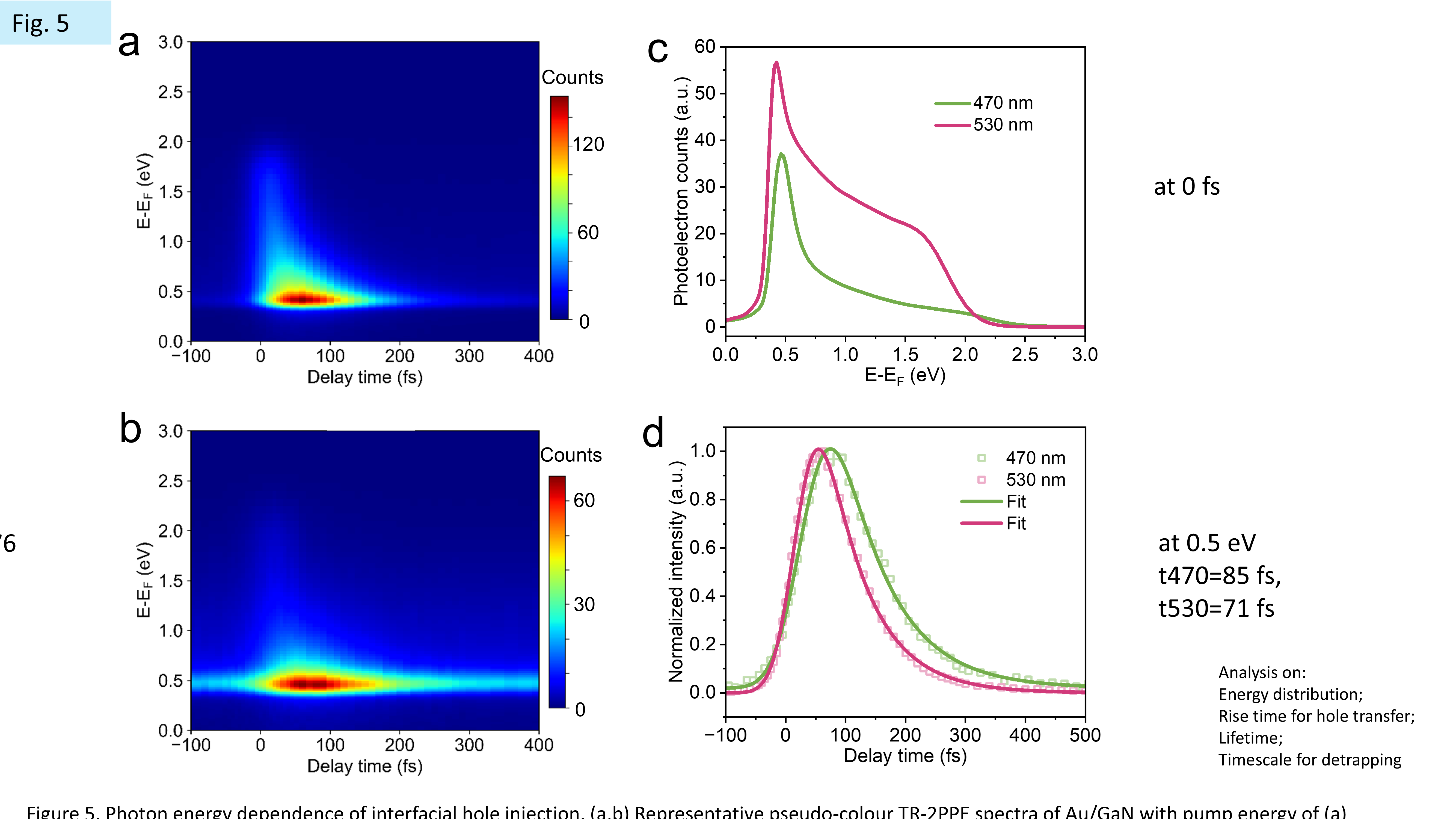


**Figure S15.** Photon energy dependence of ultrafast interfacial hole injection. (a,b) Representative pseudo-colour tr-2PPE spectra of Au/GaN with pump energy of (a) 2.34 eV (530 nm) and (b) 2.64 eV (470 nm). All spectra were measured at a probe energy of 4.49 eV (276 nm). (c) 2PPE spectra of Au/GaN excited by 470 nm and 530 nm at pump-probe delay time of 0 fs. (d) Photoelectron intensity at energy of 0.5 eV as a function of delay time for Au/GaN excited by different photon energies. The green and purple lines are fits to an exponential decay to account for the time constants.


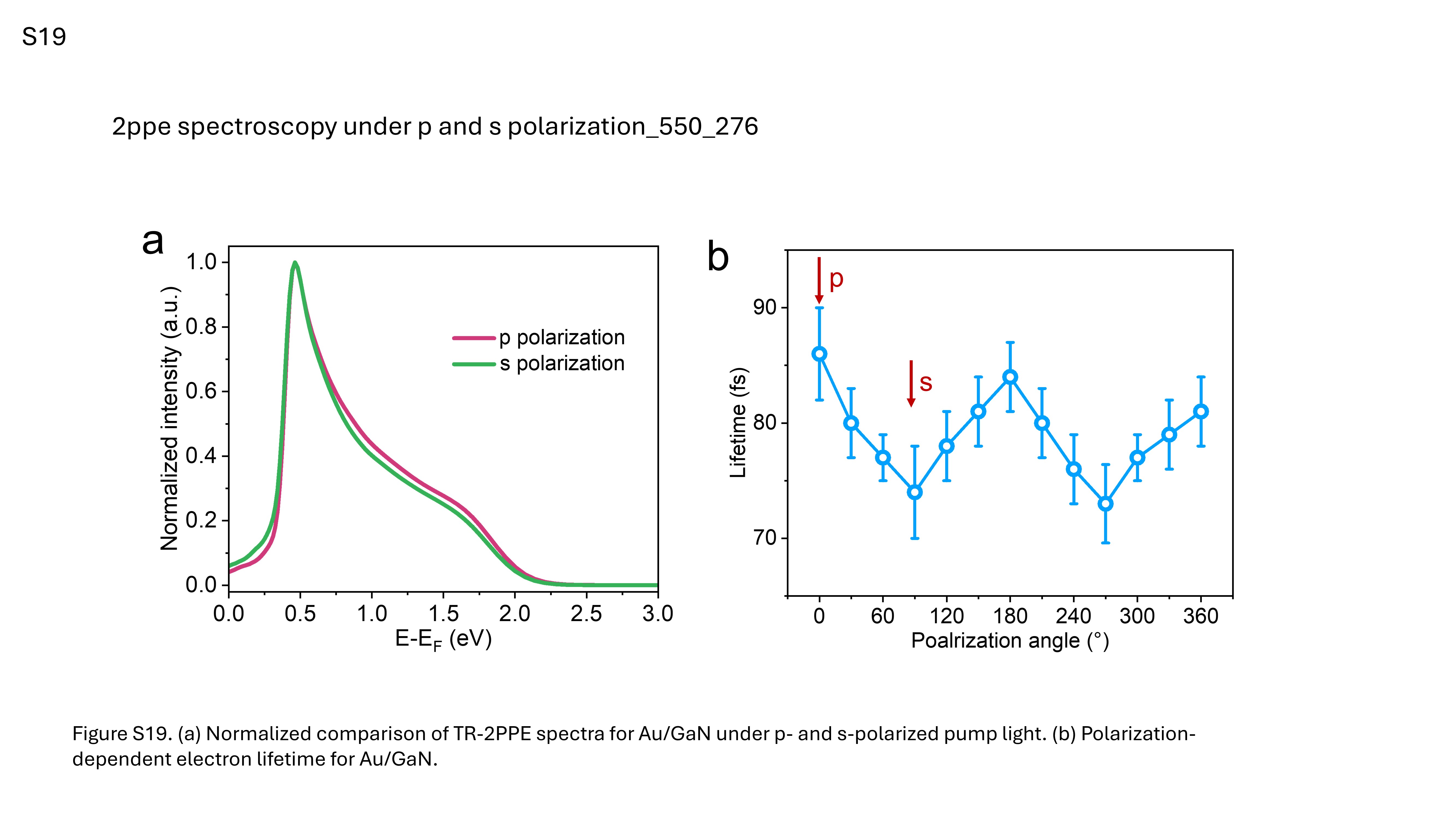


**Figure S16.** (a) Normalized comparison of tr-2PPE spectra for Au/GaN under p- and s-polarized pump excitation. (b) Polarization angle-dependent electron lifetimes for Au/GaN.


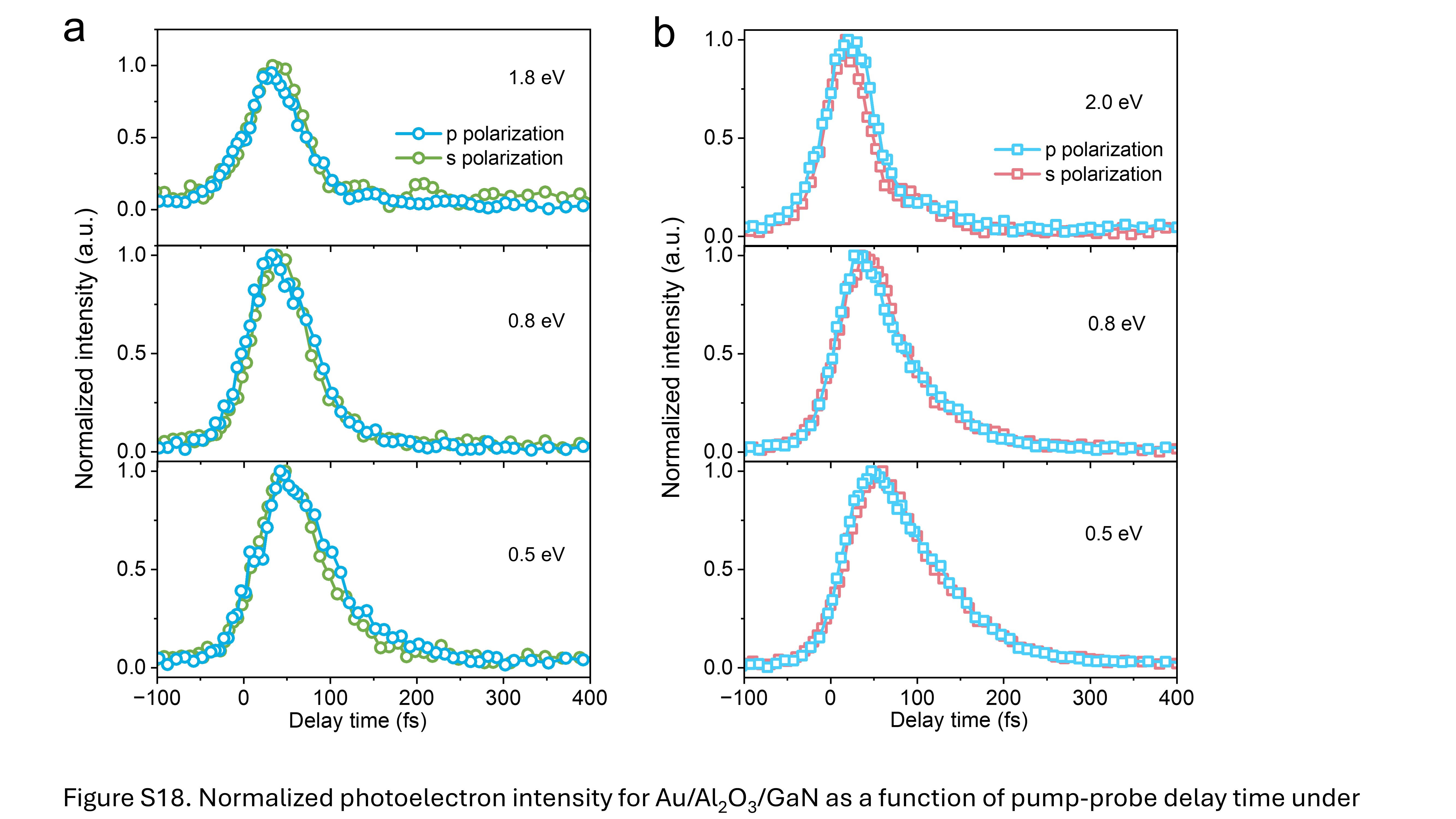


**Figure S17.** Normalized photoelectron intensity as a function of pump-probe delay time for Au/Al_2_O_3_/GaN under p- and s-polarized pump light with photon energies of (a) 2.25 eV (550 nm) and (b) 2.64 eV (470 nm) at different energies of excited electrons.

# **References**

1. S. Tan; A. Argondizzo; J. Ren; L. Liu; J. Zhao; H. Petek. "Plasmonic coupling at a metal/semiconductor interface," *Nature Photonics* 11 (2017): 806-812.

2. X. Shi; K. Ueno; T. Oshikiri; Q. Sun; K. Sasaki; H. Misawa. "Enhanced water splitting under modal strong coupling conditions," *Nature Nanotechnology* 13 (2018): 953-958.

3. C. Trovatello; F. Katsch; N. J. Borys; M. Selig; K. Yao; R. Borrego-Varillas; F. Scotognella; I. Kriegel; A. M. Yan; A. Zettl; et al. "The ultrafast onset of exciton formation in 2D semiconductors," *Nature Communications* 11 (2020): 5277.

4. S. H. Xu; H. S. Wu; X. Q. Dai; W. P. Lau; L. X. Zheng; M. H. Xie; S. Y. Tong. "Direct observation of a Ga adlayer on a GaN(0001) surface by LEED Patterson inversion," *Physical Review B* 67 (2003): 125409.

5. M. P. D. Seah, W. A. . "Quantitative Electron Spectroscopy of Surfaces: A Standard Data Base for Electron Inelastic Mean Free Paths in Solids," *Surface and Interface Analysis*1 (1979): 2-11.
